# Demand for longer quarantine period among common and uncommon COVID-19 infections: a scoping review

**DOI:** 10.1186/s40249-021-00847-y

**Published:** 2021-04-26

**Authors:** Zhi-Yao Li, Yu Zhang, Liu-Qing Peng, Rong-Rong Gao, Jia-Rui Jing, Jia-Le Wang, Bin-Zhi Ren, Jian-Guo Xu, Tong Wang

**Affiliations:** 1 Department of Health Statistics and Epidemiology, School of Public Health, Collaborative Innovation Center of Reverse Microbial Etiology, Shanxi Medical University, 56 Xinjiannanlu Street, Yingze District, Taiyuan, 030001 Shanxi People’s Republic of China; 2 Shanxi Provincial Center for Disease Control and Prevention, Taiyuan, 030001 People’s Republic of China; 3 Shanxi Provincial Key Laboratory of Major Infectious Disease Pandemic Response, Taiyuan, 030001 People’s Republic of China; 4 State Key Laboratory of Infectious Disease Prevention and Control, National Institute for Communicable Disease Control and Prevention, Chinese Center for Disease Control and Prevention, Beijing, 102206 People’s Republic of China; 5 Institute of Public Health, Nankai University, Tianjing, 300350 People’s Republic of China

**Keywords:** COVID-19, Quarantine duration, Incubation period, Asymptomatic infections, Presymptomatic infection, Recurrent positive

## Abstract

**Background:**

As one of the non-pharmacological interventions to control the transmission of COVID-19, determining the quarantine duration is mainly based on the accurate estimates of the incubation period. However, patients with coarse information of the exposure date, as well as infections other than the symptomatic, were not taken into account in previously published studies. Thus, by using the statistical method dealing with the interval-censored data, we assessed the quarantine duration for both common and uncommon infections. The latter type includes the presymptomatic, the asymptomatic and the recurrent test positive patients.

**Methods:**

As of 10 December 2020, information on cases have been collected from the English and Chinese databases, including Pubmed, Google scholar, CNKI (China National Knowledge Infrastructure) and Wanfang. Official websites and medias were also searched as data sources. All data were transformed into doubly interval-censored and the accelerated failure time model was applied. By estimating the incubation period and the time-to-event distribution of worldwide COVID-19 patients, we obtain the large percentiles for determining and suggesting the quarantine policies. For symptomatic and presymptomatic COVID-19 patients, the incubation time is the duration from exposure to symptom onset. For the asymptomatic, we substitute the date of first positive result of nucleic acid testing for that of symptom onset. Furthermore, the time from hospital discharge or getting negative test result to the positive recurrence has been calculated for recurrent positive patients.

**Results:**

A total of 1920 laboratory confirmed COVID-19 cases were included. Among all uncommon infections, 34.1% (*n* = 55) of them developed symptoms or were identified beyond fourteen days. Based on all collected cases, the 95th and 99th percentiles were estimated to be 16.2 days (95% CI 15.5–17.0) and 22.9 days (21.7‒24.3) respectively. Besides, we got similar estimates based on merely symptomatic and presymptomatic infections as 15.1 days (14.4‒15.7) and 21.1 days (20.0‒22.2).

**Conclusions:**

There are a certain number of infected people who require longer quarantine duration. Our findings well support the current practice of the extended active monitoring. To further prevent possible transmissions induced and facilitated by such infectious outliers after the 14-days quarantine, properly prolonging the quarantine duration could be prudent for high-risk scenarios and in regions with insufficient test resources.

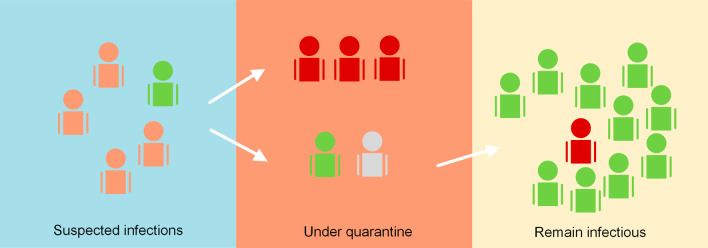

**Supplementary Information:**

The online version contains supplementary material available at 10.1186/s40249-021-00847-y.

## Background

The COVID-19 spreads worldwide rapidly, which has caused eighty million infections and nearly two million fatalities so far [1, 2]. The 14-days quarantine strategy recommended by World Health Organization is widely conducted in most countries and has already been proved effective in restraining the spreading [3, 4].

However, the 14-days duration of surveillance along with regular reverse transcription polymerase chain reaction (RT-PCR) tests was not capable of identifying all COVID-19 carriers. Recent outbreaks occurred in several countries indicated that the longer quarantine time is certainly needed for a number of infected people [5–7]. Thus, the local governments of cities in China soon proclaimed a new strategy extending formal isolation duration to 14 + 7 days in order to prevent their further transmission [8–10]. Similarly, the Ministry of Health and Family Welfare (MoHFW) of India also suggested a longer home isolation for close contacts [11].

In regions with insufficient test facilities, the quarantine policy merely depends on whether they have symptom during the isolation, while patients with longer incubation period, including the presymtpomatic ones (cases definition see Additional file 1), may become the potential source of infection after the discontinuation of quarantine. Likewise, some of asymptomatic and recovered patients require more than fourteen days to accumulate enough virus load for nasopharyngeal virus detection. Such longer period will result in temporary false negative test results during the 14-days quarantine time or hospitalization and recurrently test positive afterwards [12, 13]. These infectious outliers posed a higher risk than others to the infection control [14, 15]. Of note, the increasing number of them can invalidate the quarantine policy and finally lead to a mandatory lockdown aimed to control the spreading [16]. Yet, a quantification of a safer end limit is still lacking.

Defining the length for quarantine is mainly based on the precise estimation of the incubation period, namely the time elapsing between infection and symptom onset. To be specific, large percentiles (i.e. the 95th, 97.5th and 99th percentile) of the incubation period distribution are vital in establishing the optimal duration for quarantine [17]. In the early stage of the COVID-19 pandemic, many of the studies endorsed 14-days measures based on limited data, and tended to enroll cases with a short incubation period more frequently than other patients [18, 19], which may underestimate the proportion of patients with longer incubation period, as well as their demand for the extended quarantine duration. Although studies published later with a larger sample size reported higher 95th and 97.5th percentiles estimates [20, 21], the asymptomatic and recurrent positive cases were still not being taken into account, which introduced the bias and uncertainty into the quarantine policy making when applying such estimates as references.

To overcome the aforementioned deficiencies, we collected individual data reported by the central and local health authorities of China, and extracted information of all kinds of infections from literature published as of December 10, 2020. We aimed to summarize demographic and epidemiological characteristics among both common and uncommon infections of COVID-19, and evaluate the effectiveness of current quarantine policies by estimating the distribution of key time-to-event parameters and their large percentiles.

## Methods

### Data source and inclusion criteria

This scoping review was conducted following the recommendations of Preferred Reporting Items for Systematic Reviews and Meta-Analyses extension for Scoping reviews (PRISMA-ScR) [22]. Large English literature database (i.e. Pubmed and Google scholar), as well as preprints platforms (e.g. medRxiv) were searched to identify researches with individual data by using the “COVID-19” term and its common variations (see Additional file 1). We also searched these terms in Chinese databases, including CNKI (China National Knowledge Infrastructure, https://www.cnki.net/) [23] and Wanfang https://www.wanfangdata.com.cn/index.html. [24], since there are a number of studies published exclusively in Chinese. Searches were completed on December 10, 2020 and citations inside each article were also identified as supplements.

Additionally, daily information regarding newly confirmed cases were updated by Chinese central and local governments on their respective website, including Hong Kong, Macau and Taiwan Province, which provided characteristics and exposure histories of emerged infected patients. We also searched relevant terms on social media, including Baidu and Weibo, since the National health commission of China held online conferences during each transmission events. We manually screened such information released before December 10, 2020, to collect individual information of infected people confirmed by RT-PCR test.

To calculate the incubation or other period of each patient, eligible reports and studies should contain individual data with the following information: (1) for symptomatic and officially identified presymptomatic infections, the time of exposure and symptom onset; (2) for officially identified asymptomatic infections, the time of the exposure and the first positive test result. For patients once tested negative and recurrently tested positive during or after the isolation, we marked down their information throughout the infection course. Other characteristics as sex, age, and location of patients were also extracted.

Six listed researchers and additional four assistants were involved in data collection. These assistants first screened the reports whether contained individual data, meanwhile researchers removed duplicates, excluded non-English and non-Chinese literature from total searched studies after checking their titles and abstracts. Second, qualified reports were submitted to researchers for another scrutiny. We reviewed the full text including figures and tables of each study and report, and enrolled them according to whether they met the inclusion criteria above. Third, we divided eligible reports and studies into ten parts according to their published time, and each researchers and assistants retrieved individual data independently from the assigned part in order to avoid duplicate collection. Discussions and cross-checking were performed if there were disagreements, and consensus results were reserved to ensure consistency and precision.

### Statistical analysis

We summarized the demographic and epidemiology characteristics of all confirmed cases stratified by different kind of infections. *χ*^2^ test was used to compare the age, sex and location structure of symptomatic and other cases.

For symptomatic and presymptomatic patients, we use their time of exposure and symptom onset to estimating their incubation period. Similarly, we substitute the date of firstly test positive for the lower bound for asymptomatic infections. In terms of recurrent positive cases, we also estimate the time from hospital discharge or the first negative to the recurrence of positive, and each of hospital readmission events was documented for patients discharged more than once.

Due to different measurement methods and the population characteristics of each study, the collected individual-level data appeared in several forms, including single-interval censored data and exactly measured data (see details in Additional file 1). Therefore, we conducted a data classification and transformed all data to be double interval-censored as the optimal form for obtaining the most precise estimates [25]. We applied the accelerated failure time model to estimate the time-to-event distribution and important percentiles (i.e. the 50%, 95%, 97.5% and 99% percentile) of total included infections with three distributions (i.e. log-normal, Weibull, and gamma), and selected the best fit one based on the minimum Akaike Information Criterion. Confidence intervals for each estimated percentile were generated by using Bootstrapped and Markov chain Monte Carlo methods.

To examine the difference between using the date of the first negative testing result and hospital discharge as the starting time for the recurrent positive patients who contain them both, we separately estimated the time-to-event distribution by applying each date for sensitivity analysis. According to its original definition, the incubation period distribution was also being estimated based on mere symptomatic and presymptomatic cases in order to be comparable with previous studies. All analyses were conducted by using R software (v4.0.2, R Foundation; Vienna, Austria) with coarseDataTools, tidyverse, and lubridate packages.

## Results

**Fig. 1:**
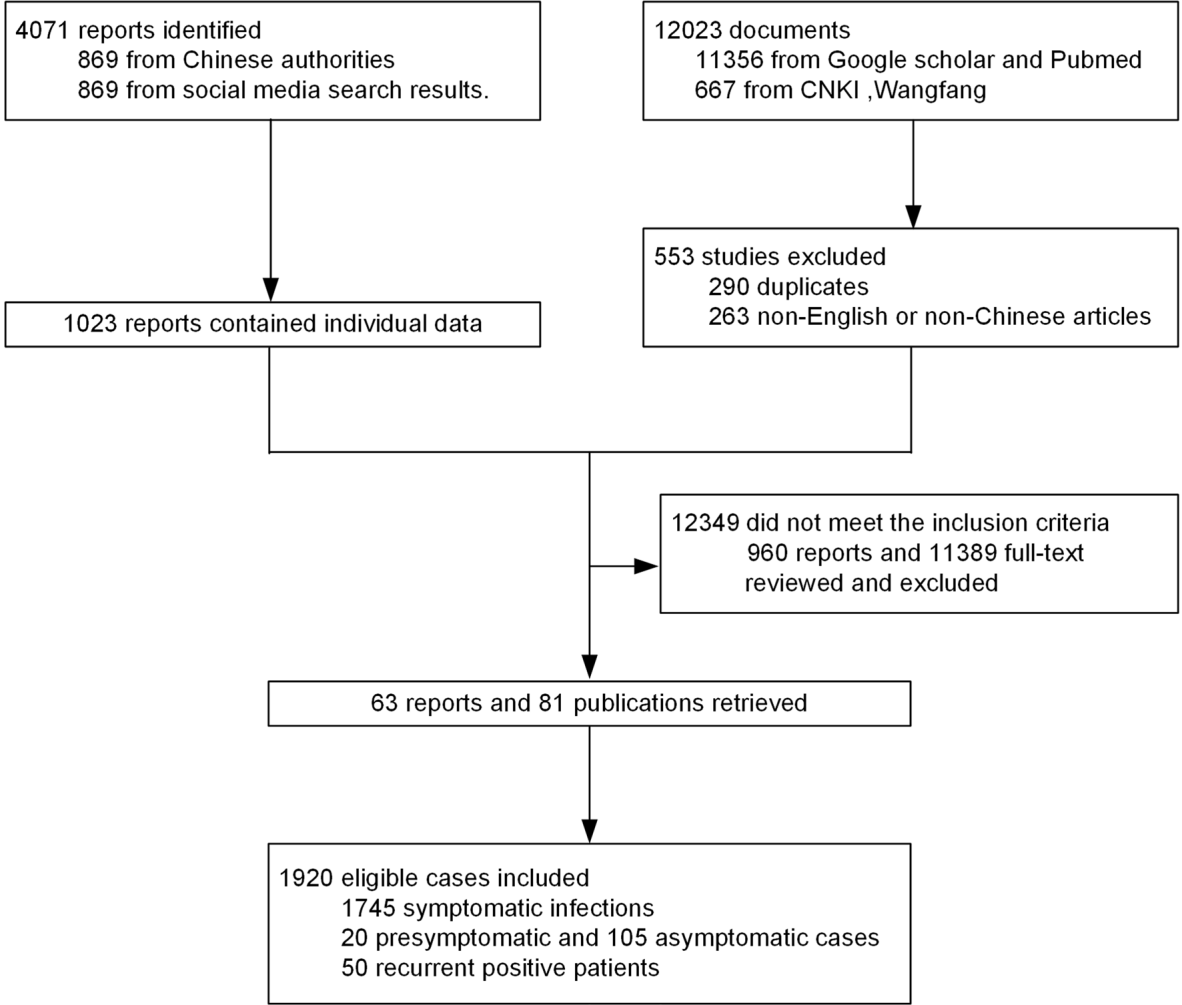
The flowchart of cases inclusion from reports and publications

We initially obtained 4071 reports and 12 023 researches as of December 10, 2020. Finally, 63 reports and 81 publications met the selection criteria identified by four reviewers. In total, 1920 laboratory confirmed cases with individual data were included and transformed before we fit them into the model. There were 1745 symptomatic cases, 20 and 105 for presymptomatic and asymptomatic infections respectively, and 50 recurrent positive patients confirmed by local health authorities in the mainland of China and other regions and countries (Fig. 1). Among all included cases in this study, 933 (48.6%) are male, and the age of total cases ranged from 34-days to 93 years old. Overall, there was no significant demographic difference between symptomatic patients and others, and no correlation was found between the age and the time-to-event (Table 1).

**Table 1:** Characteristics of common symptomatic patients and uncommon infections included in this scoping review

Characteristics	All (*n* = 1920)	Symptomatic (*n* = 1745)	Uncommon infections			
			Presymptomatic (*n* = 20)	Asymptomatic (*n* = 105)	Recurrent positive(*n* = 50)	Total (*n* = 175)
Sex, *n* (%)						
Male	933 (48.6)	86 (49.3)	1 (5.0)	36 (34.3)	36 (72.0)	73 (41.7)
Female	707 (36.8)	664 (38.1)	1 (5.0)	31 (29.5)	11 (22.0)	43 (24.6)
Unknown	280 (14.6)	221 (12.6)	18 (90.0)	38 (36.2)	3 (6.0)	59 (33.7)
Age, years, *n* (%)						
0–14	53 (2.8)	39 (2.2)	0 (0.0)	9 (8.6)	5 (10.0)	14 (8.0)
15–64	1389 (72.3)	1302 (74.6)	2 (10.0)	48 (45.7)	37 (74.0)	87 (49.7)
≥ 65	124 (6.5)	118 (6.8)	0 (0.0)	1 (1.0)	5 (10.0)	6 (3.4)
Unknown	354 (18.4)	286 (16.4)	18 (90.0)	47 (44.7)	3 (6.0)	68 (38.9)
Location, *n* (%)						
China	1557 (81.1)	1413 (81.0)	19 (95.0)	75 (71.4)	50 (100.00)	144 (82.3)
Outside the mainland of China	118 (6.1)	105 (6.0)	1 (5.0)	12 (11.4)	0 (0.00)	13 (7.4)
Unknown	245 (12.8)	227 (13.0)	0 (0.0)	18 (17.2)	0 (0.00)	18 (10.3)

**Fig. 2:**
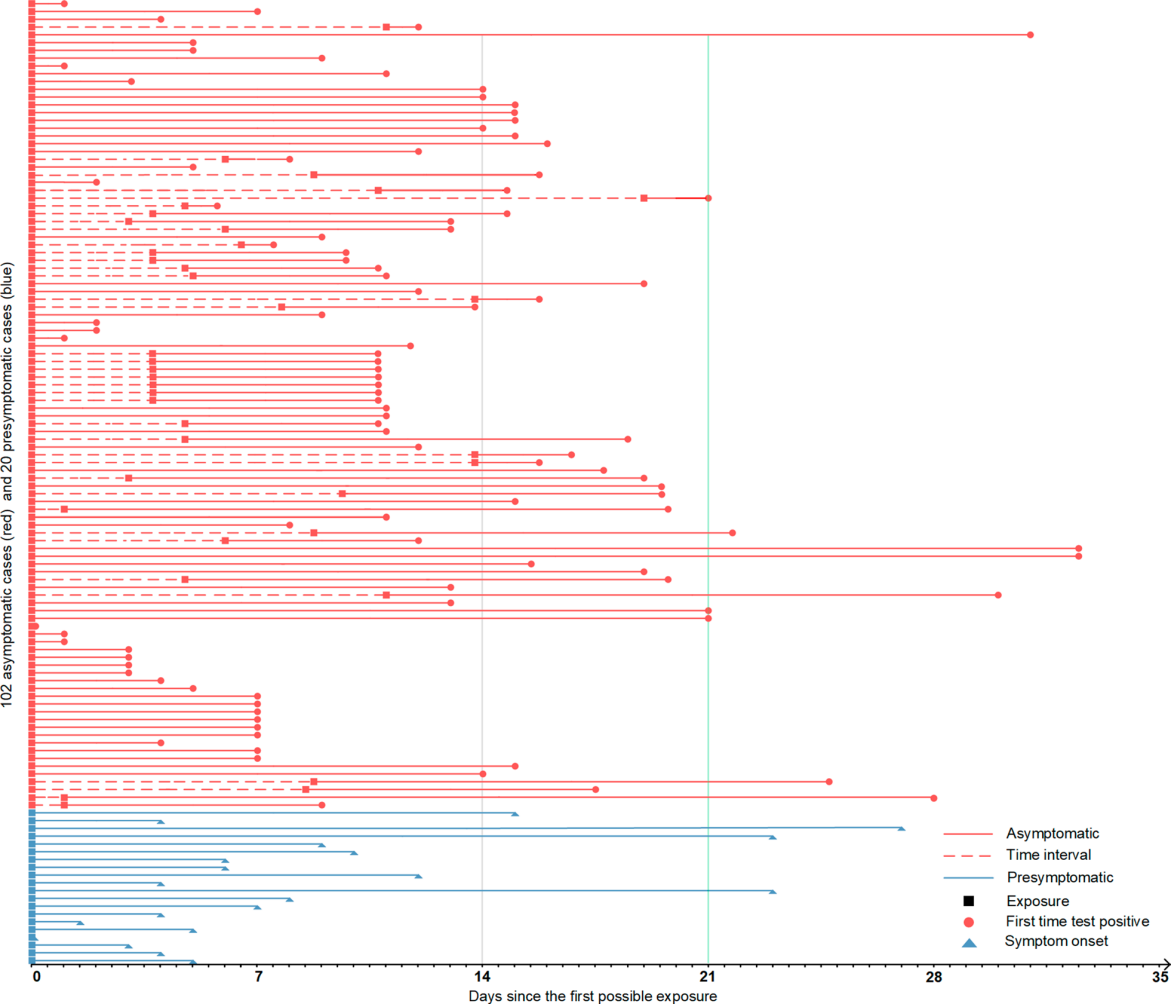
Key time to events for asymptomatic and presymptomatic infections. Each horizontal line represents one case. The date of firstly test positive for asymptomatic cases (*n* = 105; blue) is marked by solid circles, while 36.2% (*n* = 38) of them being infected within time intervals (dashed lines between squares). The symptom onset for presymptomatic cases (*n* = 20; red) is also marked by a solid triangle

**Fig. 3:**
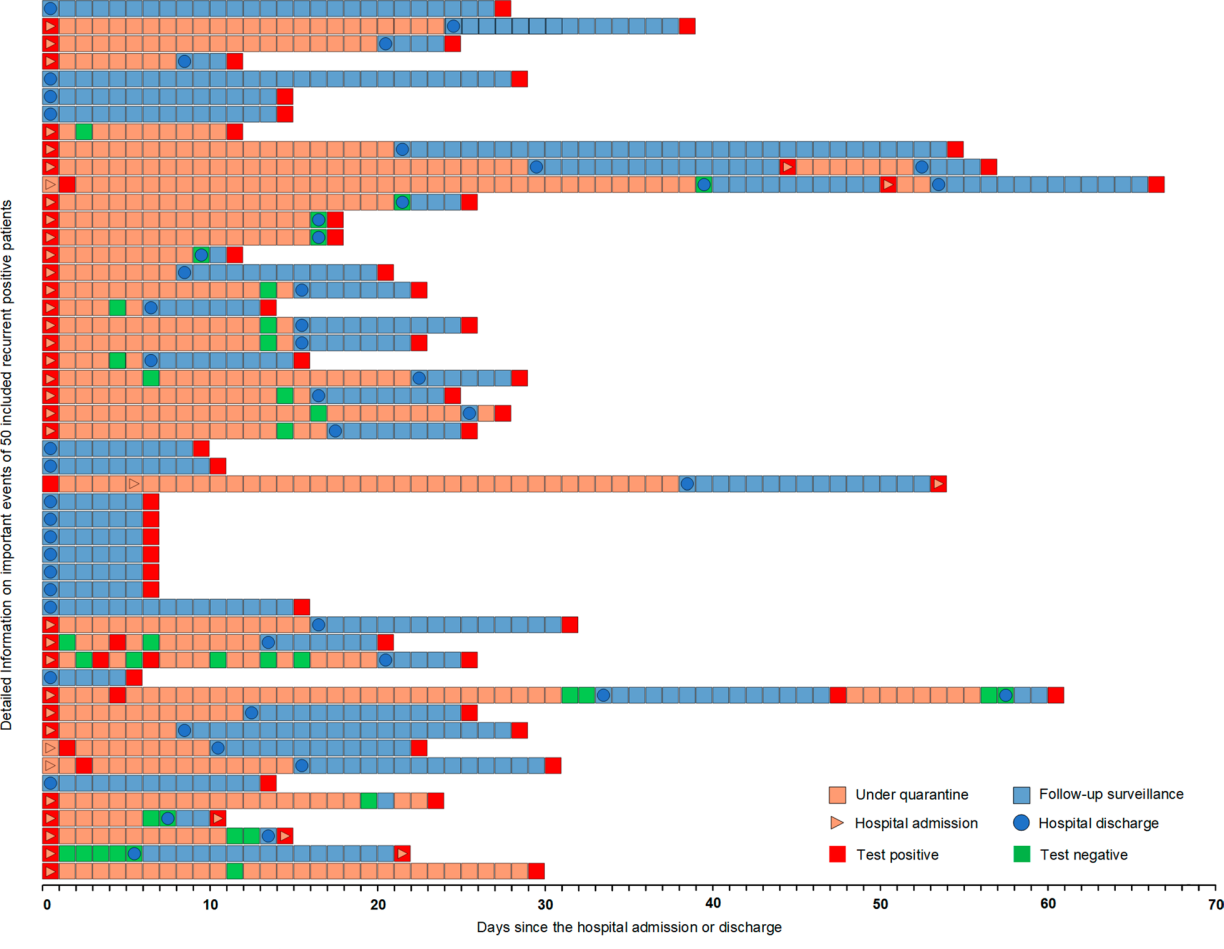
The full clinical course of 50 recurrent positive patients. Each time of testing positive and negative was marked by red and green squares respectively, and the red triangles and blue circles represent the start and the end of hospitalization

Among all included asymptomatic and presymptomatic infections, there are 32.4% (34) and 20% (4) of them required more than fourteen days to be identified or develop symptoms (using the earliest point for cases with exposure intervals), and 10 cases with the time exceeding 21-days (Fig. 2). For patients tested positive recurrently, 4 cases were identified during the hospitalization, and 4 patients were repeatedly admitted to hospital due to positive test results, with the range of 2–3 rounds. In total, 55 times of events were calculated, and 30.9% (*n* = 17) of them occurred beyond 14 days (Fig. 3).

By fitting relevant information of total 1920 cases to the model, we estimated the full distribution as well as important percentiles and it was well approximated by a Weibull distribution. The estimated median is 5.0 [95% Confidence Interval (*CI*) 4.8–5.2], and the 95th, 97.5th and 99th percentiles are 16.2 days (15.5–17.0), 19.2 days (18.2–20.2) and 22.9 days (21.7–24.3) respectively. While 9% of total cases occurred symptoms or other events beyond 14 days, according to our research, a prolonged quarantine duration of 21-days is sufficient for 98% patients. Similar results under two other parametric distributions were listed in Table 2.

**Table 2:** Estimates of important time-to-event percentiles for three parameter distributions based on patients with symptoms and total cases

Analysis	Distribution	Percentiles (95% *CI*), d				
		50	95	97.5	99	AIC
Symptomatic and presymptomatic^a^ *n* = 1765	Log-normal	4.2 (4.0‒4.4)	18.7 (17.7‒19.7)	24.9 (23.3‒26.5)	34.8 (32.1‒37.5)	8934.1
	Gamma	4.6 (4.4‒4.9)	15.5 (14.8‒16.1)	18.5 (17.6‒19.3)	22.5 (21.4‒23.6)	8722.9
	Weibull	4.8 (4.6‒5.0)	15.1 (14.4‒15.7)	17.7 (16.9‒18.6)	21.1 (20.0‒22.2)	8709.5
Total (sensitivity analysis)^b^ *n* = 1920	Log-normal	4.4 (4.2‒4.6)	20.2 (19.1‒21.2)	27.0 (25.3‒28.6)	37.8 (35.1‒40.6)	9816.0
	Gamma	4.9 (4.7‒5.1)	16.7 (15.9‒17.4)	20.0 (19.0‒20.9)	24.3 (23.1‒25.5)	9605.7
	Weibull	5.0 (4.8‒5.3)	16.3 (15.6‒17.1)	19.3 (18.4‒20.3)	23.1 (21.9‒24.4)	9599.4
Total *n* = 1920	Log-normal	4.4 (4.2‒4.6)	20.0 (18.9‒21.0)	26.7 (25.0‒28.3)	37.4 (34.7‒40.1)	9788.5
	Gamma	4.9 (4.7‒5.1)	16.5 (15.8‒17.2)	19.8 (18.9‒20.7)	24.1 (22.9‒25.3)	9579.0
	Weibull	5.0 (4.8‒5.2)	16.2 (15.5‒17.0)	19.2 (18.2‒20.2)	22.9 (21.7‒24.3)	9573.0

*CI* confidence interval

^a^By using the information of patients with symptom onset time, we obtained the incubation period estimates as its original definition

^b^Sensitivity analysis: To examine the difference between using the date of getting negative testing results and the hospital discharge for recurrent positive cases, we separately estimated the time-to-event distribution by using different dates and applied the former as the sensitivity analysis

**Fig. 4:**
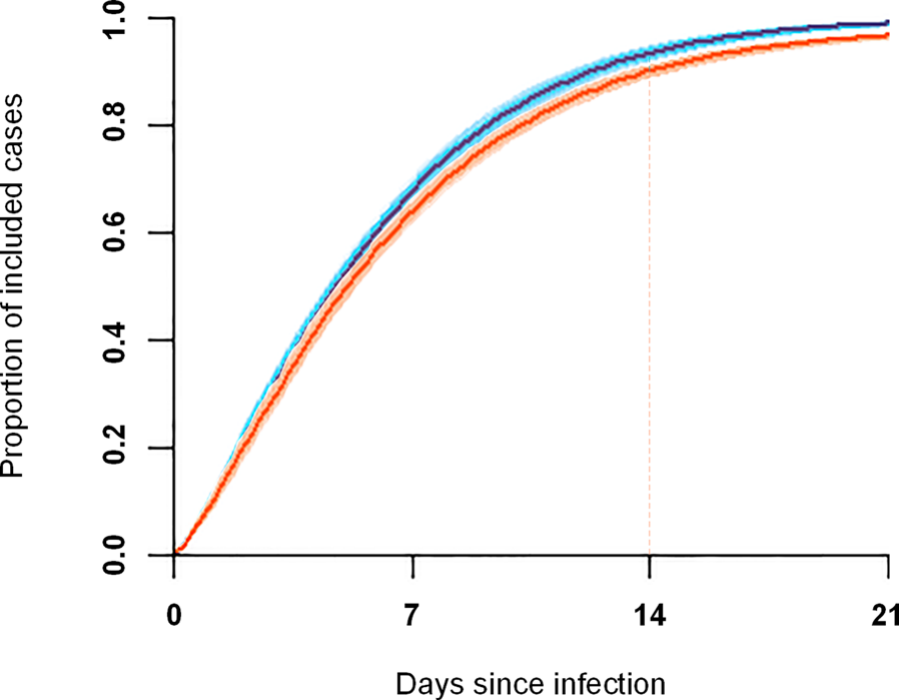
Estimating distributions of the incubation period (blue) and the time-to-event (red). The cumulative density function of the best-fitting distributions of total 1920 cases (red) and 1765 symptomatic and presymptomatic infections (blue) were separately calculated

We initially applied the date of discharge for recurrent positive patients in the analysis above. Due to different quarantine policies, the standard of the hospital discharge or the discontinuation of isolation varied from countries. In sensitivity analysis, the estimates generated by using another date were not very much different (Table 2). We also estimated the distribution of time from exposure to symptom onset based on merely symptomatic and presymptomatic patients. The median incubation period is 4.8 days (4.6–5.0), with the 95th percentile of the distribution at 15.1 days (14.4–15.7). Such estimates which are slightly smaller than the results using total cases indicates the certain need for the longer monitoring duration among asymptomatic and recurrent positive patients. Figure 4 visualizes the difference between the incubation period and the time -to-event distribution based on total cases.

## Discussion

In China, the recovered COVID-19 patients discharged from hospital as well as international passengers arriving in China would receive a 14-days centralized isolation by the law of infectious diseases control, and only could be released after they have tested negative twice at the end [26]. Similar measures are also conducted among close contacts of confirmed cases in order to separate the potential source of infection from health individuals. In this study, we have provided an assessment of current required time of quarantine duration. To the best of our knowledge, it involves the largest number of samples to date. The median of two relevant distributions in our results are 5.0 days (95% *CI* 4.8‒5.2) and 4.8 days (4.6‒5.0), lying within the range of 4–8 days reported by previous studies [27–29]. We estimated the 95th percentile among two and all kinds of infections to be 15.1 days (14.4‒15.7) and 16.2 days (15.5‒17.0) respectively, and 7%–9% (*n* = 124‒173) patients will remain infectious after 14-days isolation, while 98%-99% of total cases will be covered if the 21-days quarantine strategy is maintained. Such results of both distributions well support the current 14 + 7 period of active monitoring conducted by a number of local governments of cities in China [8–10].

Handful studies updated the estimates of large percentiles of incubation period distribution by using up-to-date data. Lu et al. [20] and Qin et al. [21] lately reported the 95th percentiles at 16.32 days (95% *CI* 15.62–17.04) and 15.1 days (14.4–15.7) respectively (other studies results see Additional file 1, Table S2), which is generally consistent with our research and indicates that the proportion of patients with longer incubation period as well as presymptomatic infections gradually increased with time.

Whether the length of the incubation period of COVID-19 varies with age or not remains uncertain. Kong [30] found that the COVID-19 incubation period was longer in older adults. By contrary, the fact that most pediatric patients tended to be mild supported the theory that the younger cases may have longer incubation periods [31]. To examine that whether the prolonged quarantine duration is suitable for all age groups, including the teenager group (0‒14 years old), the 15‒64 years old group and the group with cases aged over 65 years old, cases with age information in this study were categorized and separately analyzed. The results were shown in Additional file 1 (Table S1) and were broadly consistent with our main conclusion.

In terms of the COVID-19 presymptomatic and asymptomatic carriers, it is generally recognized that such infections posed serious challenges to intervention strategies [32]. Recent studies reported the proportion of presymptomatic and asymptomatic transmissions ranging from 15.0% to 75.9% [14, 33, 34]. As they account for 6.5% (*n* = 125) in our research, 30.4% (*n* = 38) of them required more than 14-days to be identified or develop symptoms. Properly prolonging the duration of active monitoring for possible source infector (e.g. international passenger and workers in health care centers and cold chain factories) would reduce the potential risk of transmission caused by them.

As the incidence of recurrent COVID-19 positive was reported by approximately 14.8%, the study by Azam [35] estimated the time from the last negative to the recurrent positive result by 9.8 days (95% *CI* 7.31–12.22). Despite the insufficient time for patients to accumulate enough virus for nasopharyngeal RT-PCR test, other factors including inappropriate sampling procedures and low sensitivity of test facilities may also result in temporarily negative test [36, 37]. In this study, 30.8% of total 55 times recurrent positive events occurred beyond 14-days isolation, and the longest time of virus shedding is 66 days after the first admission to hospital. As its infectiousness remain uncertain, such results suggested that more data is required to elucidate the possibility of infectious individuals with prolonged or recurrent RNA positivity, and more attention should be paid to preventing potential transmission induced by them. To the best of our knowledge, it is the first attempt to assess the required follow-up surveillance for recurrently positive patients that can also hinder control efforts.

Several limitations of the present study exist. Infections reports updated in other countries as well as studies published in languages other than English or Chinese were not included on account of the limits of languages. In addition, we made conservative assumptions on estimating the optimal quarantine duration among asymptomatic infections. Despite the fact that the active contact tracing and testing may truncate the time between exposure to detection, a number of them will develop symptom later [38, 39]. Thus, the actual result could be probably longer than current estimates by using their symptom onset.

## Conclusions

This study provides evidence that the incubation period distribution of COVID-19, especially the large percentiles, can be influenced by the carriers remained infectious at the post-quarantine stage. We not only suggest a proper quarantine duration for COVID-19, but also delivers more accurate estimates of the COVID-19 incubation period, which also plays a fundamental role in estimating other epidemiological parameters and statistical prediction modeling. As infectious outliers account for a certain proportion among all kinds of infections, in this study, the results of 95th and 99th percentiles of both two distributions were estimated to exceed fourteen days. Although such duration is applicable in most cases, to further prevent possible transmissions induced and facilitated by them, especially in regions with insufficient testing resources, strengthening the quarantine measures up to 21-days could be prudent for high-risk scenarios.

## Supplementary Information

**Additional file 1:** We offered our extended information mentioned in the main text as well as cases definitions in this supplemental file. 

## Data Availability

The sources of cases information were listed in Additional file 1. All data and materials used are available from the corresponding author on reasonable request.

## Abbreviation

CI: Confidence interval
