# Supplementary material for: Demand for longer quarantine period among common and uncommon COVID-19 infections: a scoping review
Source: Infect Dis Poverty. 2021 Apr 26;10:56. doi: 10.1186/s40249-021-00847-y (PMC8072089; doi:10.1186/s40249-021-00847-y)
Supplement: Supplementary file 1 — Additional file 1: We offered our extended information mentioned in the main text as well as cases definitions in this supplemental file. [file 40249_2021_847_MOESM1_ESM.docx]

**Supplementary Appendix**

**Contents**

[Definition of infections 2](#_Toc7522)

[Age-specific estimates under their best fit distribution 3](#_Toc28932)

[Statistical analysis 4](#_Toc4146)

[Full list of Data source for individual and aggregated data 5](#_Toc4058)

[Completeness of searching terms 27](#_Toc14071)

[Previous Studies reported large percentiles of the COVID-19 incubation period distribution 28](#_Toc22098)

[References 29](#_Toc25949)

# Definition of infections

Case definitions were based on the Protocol for COVID-19 Prevention and Control of the National Health Commission of China[1]

1.Close contacts: Individuals who had contact with a symptomatic case either before or after the source case showed symptoms or with asymptomatic cases either before or after the source case tested positive by RT–PCR. included (1) those who lived together (in the same home), studied together (in the same classroom) or worked together (in close proximity or in the same room) with the source case; (2) health care staff who provided treatment or care to the case, or family members, relatives and others who took care of or visited the case, or those who had similar close contact, such as patients who shared the same hospital ward with the case; (3) those who used the same transport as the case, including care providers on the transport, accompaniers and other passengers or crew members who might have had close contact with the case; or (4) others who met the criteria for a close contact after the investigation and evaluation

2.Symptomatic infections: patients who tested positive by RT–PCR and showed symptoms before presenting to a hospital during the 2-week quarantine period as a close contact, during the hospital stay or within 4 weeks after being discharged from the hospital. An RT–PCR cycle threshold value (Ct value) of less than 37 was defined as positive, using a commercial RT–PCR kit (DAAN Gene, 20203400063).

3.Asymptomatic infections: patients who tested positive by RT–PCR and reported no symptoms before being diagnosed and did not show symptoms throughout the quarantine and treatment period and in 4 weeks after being discharged from the hospital. Of note, cases without symptoms upon diagnosis were labeled as ‘asymptomatic’ temporarily; this label was revised to ‘symptomatic’ later if the cases developed symptoms.

4.Presymptomatic infections[2]: asymptomatic patients later develop symptoms were recategorize as presymptomatic.

5.Recurrent positive patients[3][4]: (1)cases once test negative and recurrently test positive later during the hospitalization; or (2)cases test positive after the hospital and isolation discharge, without any history of contacting with confirmed cases.

# Age-specific estimates under their best fit distribution

| Age group | Percentiles (95% *Confidence Interval*) | | |
| --- | --- | --- | --- |
|  | 95% | 97.5% | 99% |
| 0‒14 (*n* = 53) | 15.3 (12.9‒17.5) | 17.3 (14.3‒20.3) | 19.8 (15.9‒23.8) |
| 15‒64 (*n* = 1389) | 14.7 (14.0‒15.3) | 17.5 (16.7‒18.4) | 21.3 (20.1‒22.3) |
| >=65 (*n* = 124) | 14.2 (12.2‒16.3) | 17.0 (14.4‒19.6) | 20.5 (17.2‒24.0) |

**Table S1**: Large percentiles estimates of age subgroup analysis.

Statistical analysis[5][6]

**
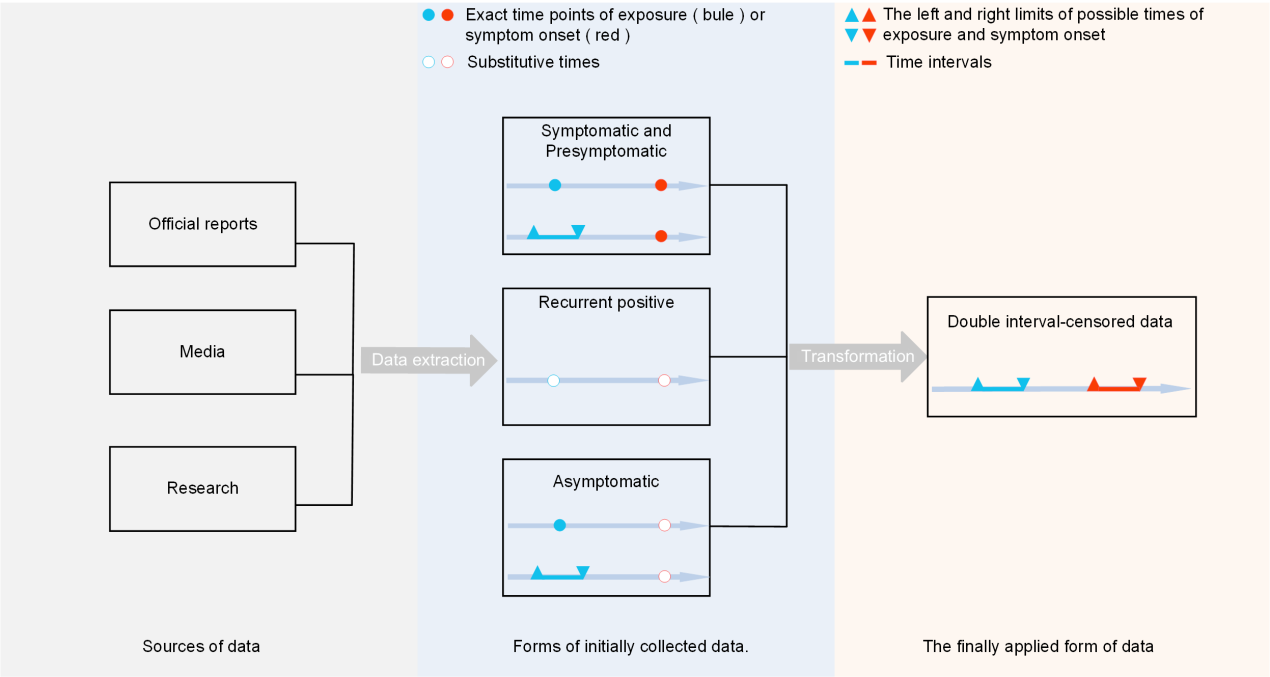
**

**Fig. S1** Data sources and transformation. Such coarse data (middle) was completely converted into the double interval-censored form by dividing one day to the beginning and the end as 00:00:00‒23:59:59, then the exact date of exposure and symptom onset can also be interval-censored.

For symptomatic and presymptomatic infections, the incubation period is defined to be the time between infection and clinical symptom onset. Let *E* and *S* be the times of the infecting exposure and symptom onset respectively. Then *T = S−E* is the incubation period. Let$f(t)$ be the probability density function (PDF) for the incubation period and the likelihood for the data is $L(\theta;X) = f(s-e)$. After the transformation including dividing one day to the beginning and the end as 00:00:00-23:59:59, both E and S of each observation were within the intervals of time. It consist of four time points (i.e. *EL, ER, SL* and *SR*) , indicating the left and right boundaries on the possible exposure and symptom onset times（Fig.S1.） The likelihood for such doubly interval censored data would be

$$L(\theta;X)=\prod_{i} \int_{{EL}_{i}}^{{ER}_{i}} \int_{{SL}_{i}}^{{SR}_{i}} g\left( e \right)f\left( s-e \right)dsde$$

, while *X* represents a data set among all symptomatic and presymptomatic cases *i* and *g(e)* is the PDF of exposure following a uniform distribution*.* We fit the PDF $f(.)$ to log-normal, Weibull, and gamma distributions.

For asymptomatic and recurrent positive patients, we also calculate the time between exposure to firstly tested positive, as well as the hospital discharge or once tested negative to later tested positive.

# Full list of Data source for individual and aggregated data

**Table .S2** Full list of data source of COVID-19.Official reports and media information as well as studies were listed bellow, including eligible and other screened ones.

| 1.Screened websites of health authorities | 1. National Health Commission of the People’s Republic of China (http://www.nhc.gov.cn)  2. Health Commission of Hubei Province (http://wjw.hubei.gov.cn)  3. Health Commission of Zhejiang Province (http://www.zjwjw.gov.cn)  4. Health Commission of Guangdong Province (http://wsjkw.gd.gov.cn)  5. Health Commission of Henan Province (http://www.hnwsjsw.gov.cn)  6. Health Commission of Hunan Province (http://wjw.hunan.gov.cn)  7. Health Commission of Anhui Province (http://wjw.ah.gov.cn)  8. Health Commission of Jiangxi Province (http://hc.jiangxi.gov.cn)  9. Health Commission of Fujian Province (http://wjw.fujian.gov.cn)  10. Health Commission of Shandong Province (http://wsjkw.shandong.gov.cn)  11. Health Commission of Shaanxi Province (http://sxwjw.shaanxi.gov.cn/)  12. Health Commission of Hebei Province (http://www.hebwst.gov.cn)  13. Health Commission of Qinghai Province (https://wsjkw.qinghai.gov.cn/)  14. Health Commission of Hainan Province (http://wst.hainan.gov.cn/swjw/index.html)  15. Health Commission of Guizhou Province (http://www.gzhfpc.gov.cn/)  16. Health Commission of Heilongjiang Province (http://wsjkw.hlj.gov.cn/)  17. Health Commission of Jilin Province (<http://wsjkw.jl.gov.cn/>)  18. Health Commission of Jiangsu Province (http://wjw.jiangsu.gov.cn/)  19. Health Commission of Gansu Province (http://wsjk.gansu.gov.cn)  20. Health Commission of Liaoning Province (http://wsjk.ln.gov.cn/)  21. Health Commission of Sichuan Province (http://wsjkw.sc.gov.cn)  22. Health Commission of Shanxi Province (http://wjw.shanxi.gov.cn/)  23. Health Commission of Yunnan Province (http://ynswsjkw.yn.gov.cn/)  24. Health Commission of Ningxia Hui Autonomous Region (http://wsjkw.nx.gov.cn)  25. Health Commission of Inner Mongolia autonomous region (http://wjw.nmg.gov.cn/)  26. Health Commission of Guangxi Zhuang Autonomous Region (http://wsjkw.gxzf.gov.cn)  27. Health Commission of Xinjiang Uygur Autonomous Region (http://www.xjhfpc.gov.cn)  28. Health Commission of Tibet Autonomous Region(http://wjw.xizang.gov.cn/)  29. Chongqing Municipal Health Commission (http://wsjkw.cq.gov.cn)  30. Shanghai Municipal Health Commission (http://wsjkw.sh.gov.cn)  31. Beijing Municipal Health Commission (http://wjw.beijing.gov.cn)  32. Tianjin Municipal Health Commission (http://wsjk.tj.gov.cn) |
| --- | --- |
| 2.Reports from social media | 1.6月10日济南新增1例境外输入无症状感染者行程轨迹公布2020/6/11济南市http://news.bandao.cn/a/377952.html  2.两例确诊病例行程轨迹公布 2020/5/15 沈阳  https://baijiahao.baidu.com/s?id=1666716406750445767&wfr=spider&for=pc  3.吉林长春16日新增1例无症状感染者行程轨迹公布 2020/5/17 长春市https://www.shobserver.com/news/detail?id=248730  4.四川成都新增1例武汉输入无症状感染者 行程轨迹公布 2020/6/19 四川成都  https://a.xcar.com.cn/bbs/thread-95348861-0.html  5.山东新增1例境外输入无症状感染者 行程轨迹公布 2020/5/24 山东省济宁市  http://m.news.cctv.com/2020/05/24/ARTI1NsvrEt9hRQJaWkhy43N200524.shtml  6.河北雄安新区公布1例无症状感染者活动轨迹 2020/6/24 河北雄安新区  http://hebei.sina.com.cn/news/2020-06-24/detail-iirczymk8784113.shtml  7.海南公布5月8日新增无症状感染者活动轨迹 2020/5/9 海南省  http://120.hinews.cn/page-78803.html  8.濮阳新增1例无症状感染者活动轨迹公布 2020/6/19 濮阳市  http://news.lyd.com.cn/system/2020/06/19/031716929.shtml  9.牡丹江公布7名无症状感染者轨迹：6人为另一人密接者  2020/5/28 牡丹江  http://news.cnnb.com.cn/system/2020/05/28/030156759.shtml  10.福建新增1例无症状感染者！轨迹公布！ 2020/5/23 福建省  http://fj.people.com.cn/n2/2020/0523/c181466-34037179.html  11.北京一无症状感染者出院28天后复阳 2020/8/15 北京  https://m.weibo.cn/status/4538079493489681?  12.北京疫情防控情况发布会：北京新增1例大连关联病例 2020/7/28 北京  https://m.weibo.cn/status/4531632985615670?  13.大连5例新增确诊行动轨迹 2020/7/30 大连市  https://m.weibo.cn/status/4532533130432198?  14.大连8例新增确诊病例轨迹公布 2020/8/3 大连市  https://m.weibo.cn/status/4533955926437527?  15.大连公布9例新增确诊详情：大连新增9例均为凯洋世界海鲜公司员工 2020/7/25  大连市 https://m.weibo.cn/2803301701/4530476260263569  16.大连新增1例本土确诊 2020/7/22 辽宁省大连市  https://m.weibo.cn/status/4529598332671290?  17.广东陆丰市南塘镇新增1例新冠确诊 2020/8/14 广东陆丰市南塘镇  https://m.weibo.cn/status/4537914841367639?  18.杭州新增1例无症状感染者 2020/8/5 杭州  https://m.weibo.cn/status/4534541706594454?  19.浙江通报新疆输入无症状感染者情况 2020/7/16 浙江  https://m.weibo.cn/status/4527245236636879?  20.铁岭公布新增病例行动轨迹 2020/7/26 铁岭市  https://m.weibo.cn/status/4530860042230481?  21.鞍山新增1例无症状感染者：鞍山新增无症状感染者行程轨迹 2020/7/24  辽宁省鞍山市 https://m.weibo.cn/2803301701/4530257832976040  22.山东省29日新增1例本土确诊病例 来自青岛市李沧区 2020/10/30 山东青岛  http://m.news.cctv.com/2020/10/30/ARTIqmy2PQOr33M1eVbRyfQY201030.shtml  23.广州花都发现1例无症状感染者 初步判断与境外输入病例相关联 2020/10/16  广州花都 http://m.news.cctv.com/2020/10/16/ARTIAeANZ6S6muZysTo9FsxA201016.shtml  24.新疆公布24日无症状感染者行动轨迹：在疏附县某制衣厂工作 2020/10/25  新疆疏附县  http://m.news.cctv.com/2020/10/25/ARTIYZHMTLd26IXvnhNzYDUC201025.shtml  25.新疆通报喀什地区疏附县疫情最新情况：新增无症状感染者61例 2020/10/31  新疆喀什 http://m.news.cctv.com/2020/10/31/ARTILl6OtaiPHiOxhaPCFiWl201031.shtml  26.青岛公布6例新增新冠肺炎确诊病例详情：均由无症状感染者转归 2020/10/14  山东青岛 http://m.news.cctv.com/2020/10/14/ARTI72kY58daNxK4rp7b6QpN201014.shtml  27.青岛新增2例新冠肺炎无症状感染者 为青岛港装卸工人 2020/9/25 山东青岛  http://m.news.cctv.com/2020/09/25/ARTIbnLrk7X16uNrvDSpdHTY200925.shtml  28.青岛新增3例新冠肺炎无症状感染者 专家组初步判断均与市胸科医院相关联  2020/10/11 山东青岛  http://m.news.cctv.com/2020/10/11/ARTIuRttVHKy8IVUXkH3AMKg201011.shtml  29.黑龙江哈尔滨市公布复阳无症状感染者行动轨迹 2020/9/25 黑龙江哈尔滨市  http://m.news.cctv.com/2020/09/25/ARTIHIZ8oAv9PBCBv3y1OjPS200925.shtml  30.黑龙江黑河新增1例确诊病例行程轨迹详情来了！ 2020/12/29 黑龙江黑河市  http://app.cctv.com/special/cportal/detail/arti/index.html?id=ArtiVBpzjZV8Wc3t57VULzqC201229&fromapp=cctvnews&version=809&allow_comment=1&allow_comment=1  31.辽宁沈阳公布新增1例本土无症状感染者详情 曾与此前确诊病例同处一室  2020/12/30 辽宁沈阳  http://app.cctv.com/special/cportal/detail/arti/index.html?id=Artibizdgyc40uemjnEqvswG201230&fromapp=cctvnews&version=809&allow_comment=1&allow_comment=1  31.北京顺义昨日7例新增确诊病例行程轨迹详情来了！ 2020/12/29 北京市顺义区  http://app.cctv.com/special/cportal/detail/arti/index.html?id=ArtiJdc0FCj73CN1qWrfsk69201229&fromapp=cctvnews&version=809&allow_comment=1&allow_comment=1  32.大连新增2例本土确诊1例本土无症状感染者 2020/12/29 辽宁大连  http://app.cctv.com/special/cportal/detail/arti/index.html?id=ArtioQuZXICyRszU88ae6PdZ201229&fromapp=cctvnews&version=809&allow_comment=1&allow_comment=1  33.成都公布昨日新增6例确诊病例详细信息 其中含1名飞行员 2020/12/25 四川成都  http://app.cctv.com/special/cportal/detail/arti/index.html?id=ArtiCxNOwPi7in2okhz8CJtE201215&fromapp=cctvnews&version=809&allow_comment=1&allow_comment=1  34.新疆吐鲁番市3例新冠肺炎无症状感染者行动轨迹公布 2020/12/12 新疆吐鲁番  http://app.cctv.com/special/cportal/detail/arti/index.html?id=ArtiUP1hhZpEoxIf9uRNET5i201212&fromapp=cctvnews&version=809&allow_comment=1&allow_comment=1  35.成都市新增4例新冠肺炎确诊病例和1名无症状感染者 病例详情公告 2020/12/8  四川成都  http://app.cctv.com/special/cportal/detail/arti/index.html?id=Arti7xbM3h5Jg3xJJnvSPbTs201208&fromapp=cctvnews&version=809&allow_comment=1&allow_comment=1  36.天津新增4例本土病例！涉疫情小区及医院已被封控！一文速览最新消息  2020/11/20 天津市  http://app.cctv.com/special/cportal/detail/arti/index.html?id=ArtinyKoMzj2Kw5yINVHU4a5201120&fromapp=cctvnews&version=809&allow_comment=1&allow_comment=1  37.澳门新增确诊1例累计7例 2020.1.28 澳门  https://m.weibo.cn/2803301701/4465641739973137  38.广东确诊1例新型冠状病毒肺炎 2020.1.20 广东  https://m.weibo.cn/2803301701/4462647154166726  39.广西确诊2例新型肺炎病例 2020.1.22 广西  https://m.weibo.cn/2803301701/4463614184053933  40.四川新增3例新型肺炎确诊病例 2020.1.22 四川  https://m.weibo.cn/status/4463601060777479?  41.辽宁确诊2例新型肺炎 2020.1.22 辽宁  https://m.weibo.cn/2803301701/4463571314096133  42.北京新增5例新型肺炎病例 2020.1.21 北京  https://m.weibo.cn/2803301701/4463308591376699  43.新疆确诊2例新型肺炎病例 2020.1.23 新疆  https://m.weibo.cn/2803301701/4463958226199785  44.辽宁新增1例确诊病例 2020.1.23 辽宁  https://m.weibo.cn/2803301701/4463860486070416  45.湖南新增5例新型肺炎确诊病例 2020.1.23 湖南  https://m.weibo.cn/2803301701/4463859325163049  46.云南新增新冠肺炎3例 2020.1.24 云南  https://m.weibo.cn/2803301701/4464403249912789  47.陕西新增2例新冠肺炎病例 2020.1.24 陕西  https://m.weibo.cn/2803301701/4464334383801217  48.内蒙古确诊首例新冠肺炎 2020.1.24 内蒙古  https://m.weibo.cn/2803301701/4464216926774457  49.速扩！急寻29日南宁病例同行人员 2020.1.30 广西南宁  https://m.weibo.cn/2803301701/4466553040834518  50.天津新增确诊2例 2020.1.29 天津  https://m.weibo.cn/2803301701/4466058985822641  51.一场同学会6人确诊 2020.1.29 合肥  https://m.weibo.cn/2803301701/4466035836911415  52.吉林新增1例累计9例 2020.1.29 吉林  https://m.weibo.cn/2803301701/4465996586753814  53.天津新增1例确诊病例 2020.1.28 天津  https://m.weibo.cn/2803301701/4465675684706033  54.吉林新增确诊病例2例 2020.1.28 吉林  https://m.weibo.cn/2803301701/4465644612983049  55.紧急扩散！河北急寻16日K4094次列出同乘人 2020.1.31 河北  https://m.weibo.cn/2803301701/4466931698297086  56.急转！寻密切接触者！夫妻确诊前自驾游途径五省市 2020.1.29 辽宁  https://m.weibo.cn/2656274875/4466152011450017  57.安徽合肥发现一例无症状感染者 密切接触者达177人 2020.3.2 安徽合肥  https://society.huanqiu.com/article/9CaKrnKpGeS?bsh_bid=5582636086  58.澳门25日又新增1例新冠肺炎输入病例 活动轨迹公布 2020.3.25 澳门  http://app.cctv.com/special/cportal/detail/arti/index.html?id=ArtidCfn2kijSicv5HUIN1da200325&fromapp=cctvnews&version=809&allow_comment=1&allow_comment=1  59.国家卫健委：17日全国新增新冠肺炎确诊病例13例 武汉新增确诊1例 2020.3.18  湖北武汉  http://app.cctv.com/special/cportal/detail/arti/index.html?id=ArtijNGilKeURfkPqV4EiAfw200318&fromapp=cctvnews&version=809&allow_comment=1&allow_comment=1  60.寻找染病车船飞机乘客 2020.2.3 河北石家庄  https://m.weibo.cn/2803301701/4467832488588815  61.河北承德公布确诊病例行程轨迹 2020.2.4 河北承德  https://m.weibo.cn/2803301701/4468193903077109  62.河北衡水公布5例确诊病例轨迹 2020.2.5 河北衡水  https://m.weibo.cn/2803301701/4468505644578237  63.64岁女子多次隐瞒疫情发生地行程被立案 2020.2.5 广东深圳  https://m.weibo.cn/2803301701/4468519905266011  64.河北秦皇岛公布3例确诊病例轨迹 2020.2.5 河北秦皇岛  https://m.weibo.cn/2803301701/4468524162827766  65.呼和浩特公布确诊病例行程轨迹 2020.2.5 内蒙呼和浩特  https://m.weibo.cn/2803301701/4468573315651157  66.河北邢台张家口公布确诊病例轨迹 2020.2.6 河北邢台  https://m.weibo.cn/2803301701/4468914186295657  67.秦皇岛公布2名新增确诊病例轨迹 2020.2.7 河北秦皇岛  https://m.weibo.cn/2803301701/4469248715933661  68.唐山公布新增2例确诊病例轨迹 2020.2.7 河北唐山  https://m.weibo.cn/2803301701/4469340222544672  69.上海昨日新增1例境外输入 2020/5/27 上海  https://m.weibo.cn/2656274875/4509109083485907  70.#吉林新增本土病例行程轨迹# 转发周知！请密切接触人员及时联系疾控中心！  2020/5/24 吉林 https://m.weibo.cn/2656274875/4508042501306491  71.吉林新增2例本土确诊 2020/5/21 吉林  https://m.weibo.cn/2656274875/4507286846768753  72.上海新增1例本土病例 2020/5/20 上海  https://s.weibo.com/weibo?q=%23%E4%B8%8A%E6%B5%B7%E6%96%B0%E5%A2%9E1%E4%BE%8B%E6%9C%AC%E5%9C%9F%E7%97%85%E4%BE%8B%23&from=default  73.吉林新增本土病例4例 2020/5/20 吉林  https://s.weibo.com/weibo?q=%23%E5%90%89%E6%9E%97%E6%96%B0%E5%A2%9E%E6%9C%AC%E5%9C%9F%E7%97%85%E4%BE%8B4%E4%BE%8B%23&from=default  74.吉林新增5例本土病例均为主动筛查确诊 2020/5/19 吉林  https://s.weibo.com/weibo?q=%23%E5%90%89%E6%9E%97%E6%96%B0%E5%A2%9E5%E4%BE%8B%E6%9C%AC%E5%9C%9F%E7%97%85%E4%BE%8B%E5%9D%87%E4%B8%BA%E4%B8%BB%E5%8A%A8%E7%AD%9B%E6%9F%A5%E7%A1%AE%E8%AF%8A%23&from=default  75.#武汉新增1例为无症状感染转确诊# 2020/5/19 武汉  https://s.weibo.com/weibo?q=%23%E6%AD%A6%E6%B1%89%E6%96%B0%E5%A2%9E1%E4%BE%8B%E4%B8%BA%E6%97%A0%E7%97%87%E7%8A%B6%E6%84%9F%E6%9F%93%E8%BD%AC%E7%A1%AE%E8%AF%8A%23&from=default  76.#吉林新增2例本土确诊# 2020/5/18 吉林  https://s.weibo.com/weibo?q=%23%E5%90%89%E6%9E%97%E6%96%B0%E5%A2%9E2%E4%BE%8B%E6%9C%AC%E5%9C%9F%E7%A1%AE%E8%AF%8A%23&from=default  77.#吉林新增3例本土确诊# 2020/5/17 吉林  https://s.weibo.com/weibo?q=%23%E5%90%89%E6%9E%97%E6%96%B0%E5%A2%9E3%E4%BE%8B%E6%9C%AC%E5%9C%9F%E7%A1%AE%E8%AF%8A%23&from=default  78.#吉林市新增2例本土病例行动轨迹# 2020/5/16 吉林  https://s.weibo.com/weibo?q=%23%E5%90%89%E6%9E%97%E5%B8%82%E6%96%B0%E5%A2%9E2%E4%BE%8B%E6%9C%AC%E5%9C%9F%E7%97%85%E4%BE%8B%E8%A1%8C%E5%8A%A8%E8%BD%A8%E8%BF%B9%23&from=default  79.[【吉林新增4例本土病例详情：#2例舒兰市密接2例丰满区密接确诊#】](https://s.weibo.com/weibo?q=%232%E4%BE%8B%E8%88%92%E5%85%B0%E5%B8%82%E5%AF%86%E6%8E%A52%E4%BE%8B%E4%B8%B0%E6%BB%A1%E5%8C%BA%E5%AF%86%E6%8E%A5%E7%A1%AE%E8%AF%8A%23&from=default) 2020/5/15  吉林  https://s.weibo.com/weibo?q=%232%E4%BE%8B%E8%88%92%E5%85%B0%E5%B8%82%E5%AF%86%E6%8E%A52%E4%BE%8B%E4%B8%B0%E6%BB%A1%E5%8C%BA%E5%AF%86%E6%8E%A5%E7%A1%AE%E8%AF%8A%23&from=default  80.#辽宁新增本土确诊病例1例#【#沈阳新增1例确诊为舒兰市聚集性疫情关联病例#】  2020/5/10 辽宁 https://m.weibo.cn/2656274875/4503140630006049  81.#武汉新增确诊病例春节后未出过小区# 3月曾有症状在家服药2020/5/10 武汉  https://m.weibo.cn/2656274875/4503022364249697  82.#吉林新增11例本土确诊病例# 2020/5/10 吉林  https://m.weibo.cn/2656274875/4502949370384964  83.#哈尔滨本土聚集性病例传染链# 2020/4/18 哈尔滨  https://s.weibo.com/weibo?q=%23%E5%93%88%E5%B0%94%E6%BB%A8%E6%9C%AC%E5%9C%9F%E8%81%9A%E9%9B%86%E6%80%A7%E7%97%85%E4%BE%8B%E4%BC%A0%E6%9F%93%E9%93%BE%23&from=default |
| 3. Studies from English database | 1.Zheng H , Tan J , Ma K , et al. Changes in RT‐PCR test results and symptoms during the menstrual cycle of female individuals infected with SARS‐CoV‐2: Report of two cases[J]. Wiley Public Health Emergency Collection.Zheng H , Tan J , Ma K , et al. Changes in RT‐PCR test results and symptoms during the menstrual cycle of female individuals infected with SARS‐CoV‐2: Report of two cases[J]. Wiley Public Health Emergency Collection.  2.Zhou J , Cao Z , Wang W , et al. First patient management of COVID-19 in Changsha, China: a case report[J]. BMC Infectious Diseases, 2020, 20(1).  3.Chen Y , Tong X , Wang J , et al. High SARS-CoV-2 antibody prevalence among healthcare workers exposed to COVID-19 patients[J]. Journal of Infection, 2020.  4.Hijnen D J , Marzano A V , Eyerich K , et al. SARS-CoV-2 Transmission from Presymptomatic Meeting Attendee, Germany[J]. Emerging Infectious Diseases, 26(8).  5.Liao R J , Ji-Ke C N , Zhang T , et al. Coronavirus disease 2019 epidemic in impoverished area: Liangshan Yi autonomous prefecture as an example[J]. Infectious Diseases of Poverty, 2020, 9(1).  6.Scott S E , Zabel K , Collins J , et al. First Mildly Ill, Non-Hospitalized Case of Coronavirus Disease 2019 (COVID-19) Without Viral Transmission in the United States - Maricopa County, Arizona, 2020[J]. Clinical Infectious Diseases, 2020, 71(15).  7.Li W , Su Y Y , Zhi S S , et al. Viral shedding dynamics in asymptomatic and mildly symptomatic patients infected with SARS-CoV-2[J]. Clinical Microbiology and Infection, 2020.  8.Bhmer M M , Buchholz U , Corman V M , et al. Articles Investigation of a COVID-19 outbreak in Germany resulting from a single travel-associated primary case: a case series[J]. The Lancet Infectious Diseases, 2020, 20(8).  9.Alexandre C , Brito A D , Brito M C M D , et al. Coronavirus Pandemic Clinical laboratory and dispersion pattern of COVID-19 in a family cluster in the social-distancing period[J]. The Journal of Infection in Developing Countries, 2020, 14(9):987-993.  10.Zhang X , Wang H , Wang Y , et al. Epidemiological and clinical based study on four passages of COVID-19 patients: intervention at asymptomatic period contributes to early recovery[J]. BMC Infectious Diseases, 2020, 20(1).  11.Ye Q , Wang B , Mao J , et al. Epidemiological analysis of COVID-19 and practical experience from China[J]. Journal of Medical Virology, 2020, 92(2).  12.Zhang J , Litvinova M , Wang W , et al. Evolving epidemiology and transmission dynamics of coronavirus disease 2019 outside Hubei province, China: a descriptive and modelling study[J]. The Lancet Infectious Diseases, 2020, 20(7).  13.Su L , Ma X , Yu H , et al. The different clinical characteristics of corona virus disease cases between children and their families in China – the character of children with COVID-19[J]. Emerging Microbes and Infections, 2020, 9(1):707-713.  14.Bao F , Xiaoyun F . Clinical characteristics of 11 asymptomatic patients with COVID-19[J]. Medicina Clínica, 2020.  15.Kutsuna S , Suzuki T , Hayakawa K , et al. SARS-CoV-2 screening test for Japanese returnees from Wuhan, China, January 2020[J]. Open Forum Infectious Diseases, 2020.  16.Yin S , Peng Y , Ren Y , et al. The implications of preliminary screening and diagnosis: Clinical characteristics of 33 mild patients with SARS-CoV-2 infection in Hunan, China[J]. Journal of Clinical Virology, 2020, 128:104397.  17.Liu Z , Chu R , Gong L , et al. The assessment of transmission efficiency and latent infection period on asymptomatic carriers of SARS-CoV-2 infection[J]. International Journal of Infectious Diseases, 2020.  18.Jinila BY, Shyry PS. Transmissibility and Epidemicity of COVID-19 in India: A Case Study. Recent Pat Antiinfect Drug Discov. 2020 Sep 15. doi: 10.2174/1574891X15666200915140806.  19.Sanche S, Lin YT, Xu C et al. High Contagiousness and Rapid Spread of Severe Acute Respiratory Syndrome Coronavirus 2. Emerg Infect Dis. 2020 Jul;26(7):1470-1477. doi: 10.3201/eid2607.200282.  20.Li C, Ji F, Wang L et al. Asymptomatic and Human-to-Human Transmission of SARS-CoV-2 in a 2-Family Cluster, Xuzhou, China. Emerg Infect Dis. 2020 Jul;26(7):1626-1628. doi: 10.3201/eid2607.200718.  21.Grall I, Alloui CA, Tandjaoui-Lambiotte Y et al. Viral transmission in asymptomatic cases of SARS-CoV-2 infection. J Infect. 2020 Aug 29:S0163-4453(20)30578-8. doi: 10.1016/j.jinf.2020.08.044.  22.Ren JG, Li DY, Wang CF, Wu JH, Wang Y, Sun YJ, Zhang Q, Wang YY, Chang XJ. Positive RT-PCR in urine from an asymptomatic patient with novel coronavirus 2019 infection: a case report. Infect Dis (Lond). 2020 Aug;52(8):571-574. doi: 10.1080/23744235.2020.1766105.  23.Hu S, Wang W, Wang Y et al. Infectivity, susceptibility, and risk factors associated with SARS-CoV-2 transmission under intensive contact tracing in Hunan, China. medRxiv [Preprint]. 2020 Aug 7:2020.07.23.20160317. doi: 10.1101/2020.07.23.20160317.  24.Bi Q, Wu Y, Mei S et al. Epidemiology and transmission of COVID-19 in 391 cases and 1286 of their close contacts in Shenzhen, China: a retrospective cohort study. Lancet Infect Dis. 2020 Aug;20(8):911-919. doi: 10.1016/S1473-3099(20)30287-5.  25.Shi Q, Hu Y, Peng B et al. Effective control of SARS-CoV-2 transmission in Wanzhou, China. Nat Med. 2021 Jan;27(1):86-93. doi: 10.1038/s41591-020-01178-5.  26.Bae JM. A Chinese Case of COVID-19 Did Not Show Infectivity During the Incubation Period: Based on an Epidemiological Survey [published online ahead of print, 2020 Mar 2]. J Prev Med Public Health. 2020;53(2):67-69. doi:10.3961/jpmph.20.048  27.Gao Y, Shi C, Chen Y, et al. A cluster of the Corona Virus Disease 2019 caused by incubation period transmission in Wuxi, China. J Infect. 2020;80(6):666-670. doi:10.1016/j.jinf.2020.03.042  28.Chan JF, Yuan S, Kok KH, et al. A familial cluster of pneumonia associated with the 2019 novel coronavirus indicating person-to-person transmission: a study of a family cluster. Lancet. 2020;395(10223):514-523. doi:10.1016/S0140-6736(20)30154-9  29.Tian S, Hu N, Lou J, et al. Characteristics of COVID-19 infection in Beijing. J Infect. 2020;80(4):401-406. doi:10.1016/j.jinf.2020.02.018  30.Song R, Han B, Song M, et al. Clinical and epidemiological features of COVID-19 family clusters in Beijing, China [published online ahead of print, 2020 Apr 23]. J Infect. 2020;S0163-4453(20)30229-2. doi:10.1016/j.jinf.2020.04.018  31.Hu Z, Song C, Xu C, et al. Clinical characteristics of 24 asymptomatic infections with COVID-19 screened among close contacts in Nanjing, China. Sci China Life Sci. 2020;63(5):706-711. doi:10.1007/s11427-020-1661-4  32.Lei S, Jiang F, Su W, et al. Clinical characteristics and outcomes of patients undergoing surgeries during the incubation period of COVID-19 infection [published online ahead of print, 2020 Apr 5]. EClinicalMedicine. 2020;21:100331. doi:10.1016/j.eclinm.2020.100331  33.Liu SF, Kuo NY, Kuo HC. Comparison of the Characteristics and Outcomes of Coronavirus Disease 2019 in Different Types of Family Infections in Taiwan. J Clin Med. 2020;9(5):1527. Published 2020 May 19. doi:10.3390/jcm9051527  34.Li Q, Guan X, Wu P, et al. Early Transmission Dynamics in Wuhan, China, of Novel Coronavirus-Infected Pneumonia. N Engl J Med. 2020;382(13):1199-1207. doi:10.1056/NEJMoa2001316  35.Ki M; Task Force for 2019-nCoV. Epidemiologic characteristics of early cases with 2019 novel coronavirus (2019-nCoV) disease in Korea. Epidemiol Health. 2020;42:e2020007. doi:10.4178/epih.e2020007  36.Pan Y, Yu X, Du X, et al. Epidemiological and Clinical Characteristics of 26 Asymptomatic Severe Acute Respiratory Syndrome Coronavirus 2 Carriers. J Infect Dis. 2020;221(12):1940-1947. doi:10.1093/infdis/jiaa205  37.Xia XY, Wu J, Liu HL, Xia H, Jia B, Huang WX. Epidemiological and initial clinical characteristics of patients with family aggregation of COVID-19. J Clin Virol. 2020;127:104360. doi:10.1016/j.jcv.2020.104360  38.Guan Q, Liu M, Zhuang YJ, et al. Zhonghua Liu Xing Bing Xue Za Zhi. 2020;41(5):629-633. doi:10.3760/cma.j.cn112338-20200223-00152  39.Sanche S, Lin YT, Xu C, Romero-Severson E, Hengartner N, Ke R. High Contagiousness and Rapid Spread of Severe Acute Respiratory Syndrome Coronavirus 2. Emerg Infect Dis. 2020;26(7):1470-1477. doi:10.3201/eid2607.200282  40.Linton NM, Kobayashi T, Yang Y, et al. Incubation Period and Other Epidemiological Characteristics of 2019 Novel Coronavirus Infections with Right Truncation: A Statistical Analysis of Publicly Available Case Data. J Clin Med. 2020;9(2):538. Published 2020 Feb 17. doi:10.3390/jcm9020538  41.Backer JA, Klinkenberg D, Wallinga J. Incubation period of 2019 novel coronavirus (2019-nCoV) infections among travellers from Wuhan, China, 20-28 January 2020. Euro Surveill. 2020;25(5):2000062. doi:10.2807/1560-7917.ES.2020.25.5.2000062  42.Cai J, Sun W, Huang J, Gamber M, Wu J, He G. Indirect Virus Transmission in Cluster of COVID-19 Cases, Wenzhou, China, 2020. Emerg Infect Dis. 2020;26(6):1343-1345. doi:10.3201/eid2606.200412  43.Böhmer MM, Buchholz U, Corman VM, et al. Investigation of a COVID-19 outbreak in Germany resulting from a single travel-associated primary case: a case series [published online ahead of print, 2020 May 15]. Lancet Infect Dis. 2020;S1473-3099(20)30314-5. doi:10.1016/S1473-3099(20)30314-5  44.Pung R, Chiew CJ, Young BE, et al. Investigation of three clusters of COVID-19 in Singapore: implications for surveillance and response measures. Lancet. 2020;395(10229):1039-1046. doi:10.1016/S0140-6736(20)30528-6  45.Liu SF, Kuo NY, Kuo HC. Three Taiwan's domestic family cluster infections of coronavirus disease 2019 [published online ahead of print, 2020 Apr 28]. J Med Virol. 2020;10.1002/jmv.25949. doi:10.1002/jmv.25949  46.Li P, Fu JB, Li KF, et al. Transmission of COVID-19 in the terminal stages of the incubation period: A familial cluster. Int J Infect Dis. 2020;96:452-453. doi:10.1016/j.ijid.2020.03.027  47.Bai SL, Wang JY, Zhou YQ, et al. Zhonghua Yu Fang Yi Xue Za Zhi. 2020;54(5):491-493. doi:10.3760/cma.j.cn112150-20200204-00065  48.Ning SS, Zhang Y, Cao L, et al. Zhonghua Yu Fang Yi Xue Za Zhi. 2020;54(5):493-497. doi:10.3760/cma.j.cn112150-20200227-00201  49.Xiao WJ, Gao Q, Jin K, et al. Zhonghua Liu Xing Bing Xue Za Zhi. 2020;41(0):E033. doi:10.3760/cma.j.cn112338-20200302-00236  50.Wu WS, Li YG, Wei ZF, et al. Zhonghua Liu Xing Bing Xue Za Zhi. 2020;41(4):489-493. doi:10.3760/cma.j.cn112338-20200221-00139  51.Yang HY, Xu J, Li Y, et al. Zhonghua Liu Xing Bing Xue Za Zhi. 2020;41(5):623-628. doi:10.3760/cma.j.cn112338-20200223-00153  52.Zhang Y, Su X, Chen W, et al. Zhonghua Liu Xing Bing Xue Za Zhi. 2020;41(5):648-652. doi:10.3760/cma.j.cn112338-20200219-00121  53.Qiu YY, Wang SQ, Wang XL, et al. Zhonghua Liu Xing Bing Xue Za Zhi. 2020;41(4):494-497. doi:10.3760/cma.j.cn112338-20200221-00147  54.Zhao H, Li BS, Xia Y, et al. Zhonghua Liu Xing Bing Xue Za Zhi. 2020;41(0):E064. doi:10.3760/cma.j.cn112338-20200227-00198  55.Lauer SA, Grantz KH, Bi Q, et al. The Incubation Period of Coronavirus Disease 2019 (COVID-19) From Publicly Reported Confirmed Cases: Estimation and Application. Ann Intern Med. 2020;172(9):577-582. doi:10.7326/M20-0504  56.Song QQ, Zhao H, Fang LQ, Liu W, Zheng C, Zhang Y. Zhonghua Liu Xing Bing Xue Za Zhi. 2020;41(4):461-465. doi:10.3760/cma.j.cn112338-20200205-00069  57.Chen Y, Wang AH, Yi B, et al. Zhonghua Liu Xing Bing Xue Za Zhi. 2020;41(5):667-671. doi:10.3760/cma.j.cn112338-20200304-00251  58.Leung C. The difference in the incubation period of 2019 novel coronavirus (SARS-CoV-2) infection between travelers to Hubei and nontravelers: The need for a longer quarantine period. Infect Control Hosp Epidemiol. 2020;41(5):594-596. doi:10.1017/ice.2020.81  59.Chen J, Zhang ZZ, Chen YK, et al. The clinical and immunological features of pediatric COVID-19 patients in China [published online ahead of print, 2020 Apr 14]. Genes Dis. 2020;10.1016/j.gendis.2020.03.008. doi:10.1016/j.gendis.2020.03.008  60.Gou FX, Zhang XS, Yao JX, et al. Zhonghua Liu Xing Bing Xue Za Zhi. 2020;41(0):E032. doi:10.3760/cma.j.cn112338-20200229-00216  61.Peirlinck M, Linka K, Sahli Costabal F, Kuhl E. Outbreak dynamics of COVID-19 in China and the United States [published online ahead of print, 2020 Apr 27]. Biomech Model Mechanobiol. 2020;1-15. doi:10.1007/s10237-020-01332-5  62.Mash B. Primary care management of the coronavirus (COVID-19) [published correction appears in S Afr Fam Pract (2004). 2020 Jun 10;62(1):5144]. S Afr Fam Pract (2004). 2020;62(1):e1-e4. Published 2020 Mar 31. doi:10.4102/safp.v62i1.5115  63.Sun C, Zhang XB, Dai Y, Xu XZ, Zhao J. Zhonghua Jie He He Hu Xi Za Zhi. 2020;43(6):503-508. doi:10.3760/cma.j.cn112147-20200224-00168  64.Marschner IC. Back-projection of COVID-19 diagnosis counts to assess infection incidence and control measures: analysis of Australian data. Epidemiol Infect. 2020;148:e97. Published 2020 May 18. doi:10.1017/S0950268820001065  65.Kong W, Wang Y, Hu J, Chughtai A, Pu H; Clinical Research Collaborative Group of Sichuan Provincial People's Hospital. Comparison of clinical and epidemiological characteristics of asymptomatic and symptomatic SARS-CoV-2 infection: A multi-center study in Sichuan Province, China [published online ahead of print, 2020 May 31]. Travel Med Infect Dis. 2020;101754. doi:10.1016/j.tmaid.2020.101754  66.Jia J, Hu X, Yang F, et al. Epidemiological Characteristics on the Clustering Nature of COVID-19 in Qingdao City, 2020: A Descriptive Analysis [published online ahead of print, 2020 Mar 31]. Disaster Med Public Health Prep. 2020;1-5. doi:10.1017/dmp.2020.59  67.He W, Yi GY, Zhu Y. Estimation of the basic reproduction number, average incubation time, asymptomatic infection rate, and case fatality rate for COVID-19: Meta-analysis and sensitivity analysis [published online ahead of print, 2020 May 29]. J Med Virol. 2020;10.1002/jmv.26041. doi:10.1002/jmv.26041  68.Wu JT, Leung K, Leung GM. Nowcasting and forecasting the potential domestic and international spread of the 2019-nCoV outbreak originating in Wuhan, China: a modelling study [published correction appears in Lancet. 2020 Feb 4;:]. Lancet. 2020;395(10225):689-697. doi:10.1016/S0140-6736(20)30260-9  69.Wu HP, Li BF, Chen X, et al. Zhongguo Dang Dai Er Ke Za Zhi. 2020;22(5):419-424.  70.Wang P, Lu JA, Jin Y, Zhu M, Wang L, Chen S. Statistical and network analysis of 1212 COVID-19 patients in Henan, China. Int J Infect Dis. 2020;95:391-398. doi:10.1016/j.ijid.2020.04.051  71.Nie X, Fan L, Mu G, et al. Epidemiological Characteristics and Incubation Period of 7015 Confirmed Cases With Coronavirus Disease 2019 Outside Hubei Province in China. J Infect Dis. 2020;222(1):26-33. doi:10.1093/infdis/jiaa211  72.Lauer SA, Grantz KH, Bi Q, et al. The Incubation Period of Coronavirus Disease 2019 (COVID-19) From Publicly Reported Confirmed Cases: Estimation and Application. Ann Intern Med. 2020;172(9):577-582. doi:10.7326/M20-0504  73.Sun L, Shen L, Fan J, et al. Clinical features of patients with coronavirus disease 2019 from a designated hospital in Beijing, China [published online ahead of print, 2020 May 5]. J Med Virol. 2020;10.1002/jmv.25966. doi:10.1002/jmv.25966  74.Cheng HY, Jian SW, Liu DP, et al. Contact Tracing Assessment of COVID-19 Transmission Dynamics in Taiwan and Risk at Different Exposure Periods Before and After Symptom Onset [published online ahead of print, 2020 May 1]. JAMA Intern Med. 2020;e202020. doi:10.1001/jamainternmed.2020.2020  75.Jiang X, Rayner S, Luo MH. Does SARS-CoV-2 has a longer incubation period than SARS and MERS?. J Med Virol. 2020;92(5):476-478. doi:10.1002/jmv.25708  76.Li Q, Guan X, Wu P, et al. Early Transmission Dynamics in Wuhan, China, of Novel Coronavirus-Infected Pneumonia. N Engl J Med. 2020;382(13):1199-1207. doi:10.1056/NEJMoa2001316  77.Qian GQ, Yang NB, Ding F, et al. Epidemiologic and clinical characteristics of 91 hospitalized patients with COVID-19 in Zhejiang, China: a retrospective, multi-centre case series. QJM. 2020;113(7):474-481. doi:10.1093/qjmed/hcaa089  78.Ganyani T, Kremer C, Chen D, et al. Estimating the generation interval for coronavirus disease (COVID-19) based on symptom onset data, March 2020. Euro Surveill. 2020;25(17):2000257. doi:10.2807/1560-7917.ES.2020.25.17.2000257  79.Zhang J, Litvinova M, Wang W, et al. Evolving epidemiology and transmission dynamics of coronavirus disease 2019 outside Hubei province, China: a descriptive and modelling study. Lancet Infect Dis. 2020;20(7):793-802. doi:10.1016/S1473-3099(20)30230-9  80.Xiao Z, Xie X, Guo W, et al. Examining the incubation period distributions of COVID-19 on Chinese patients with different travel histories. J Infect Dev Ctries. 2020;14(4):323-327. Published 2020 Apr 30. doi:10.3855/jidc.12718  81.Linton NM, Kobayashi T, Yang Y, et al. Incubation Period and Other Epidemiological Characteristics of 2019 Novel Coronavirus Infections with Right Truncation: A Statistical Analysis of Publicly Available Case Data. J Clin Med. 2020;9(2):538. Published 2020 Feb 17. doi:10.3390/jcm9020538  82.Wu Z, McGoogan JM. Characteristics of and Important Lessons From the Coronavirus Disease 2019 (COVID-19) Outbreak in China: Summary of a Report of 72 314 Cases From the Chinese Center for Disease Control and Prevention [published online ahead of print, 2020 Feb 24]. JAMA. 2020;10.1001/jama.2020.2648. doi:10.1001/jama.2020.2648  83.Chan JF, Yuan S, Kok KH, et al. A familial cluster of pneumonia associated with the 2019 novel coronavirus indicating person-to-person transmission: a study of a family cluster. Lancet. 2020;395(10223):514-523. doi:10.1016/S0140-6736(20)30154-9  84.Huang C, Wang Y, Li X, et al. Clinical features of patients infected with 2019 novel coronavirus in Wuhan, China [published correction appears in Lancet. 2020 Jan 30;:]. Lancet. 2020;395(10223):497-506. doi:10.1016/S0140-6736(20)30183-5  85.Yang Y, Lu Q, Liu M, et al. Epidemiological and clinical features of the 2019 novel coronavirus outbreak in China[J]. medRxiv, 2020.  86.Guan WJ, Ni ZY, Hu Y, et al. Clinical Characteristics of Coronavirus Disease 2019 in China. N Engl J Med. 2020;382(18):1708-1720. doi:10.1056/NEJMoa2002032  87.Backer JA, Klinkenberg D, Wallinga J. Incubation period of 2019 novel coronavirus (2019-nCoV) infections among travellers from Wuhan, China, 20-28 January 2020. Euro Surveill. 2020;25(5):2000062. doi:10.2807/1560-7917.ES.2020.25.5.2000062  88.Munster VJ, Koopmans M, van Doremalen N, van Riel D, de Wit E. A Novel Coronavirus Emerging in China - Key Questions for Impact Assessment. N Engl J Med. 2020;382(8):692-694. doi:10.1056/NEJMp2000929  89.Zhang MQ, Wang XH, Chen YL, et al. Zhonghua Jie He He Hu Xi Za Zhi. 2020;43(3):215-218. doi:10.3760/cma.j.issn.1001-0939.2020.03.015  90.Bae JM. A Chinese Case of COVID-19 Did Not Show Infectivity During the Incubation Period: Based on an Epidemiological Survey [published online ahead of print, 2020 Mar 2]. J Prev Med Public Health. 2020;53(2):67-69. doi:10.3961/jpmph.20.048  91.Gao Y, Shi C, Chen Y, et al. A cluster of the Corona Virus Disease 2019 caused by incubation period transmission in Wuxi, China. J Infect. 2020;80(6):666-670. doi:10.1016/j.jinf.2020.03.042  92.Chan JF, Yuan S, Kok KH, et al. A familial cluster of pneumonia associated with the 2019 novel coronavirus indicating person-to-person transmission: a study of a family cluster. Lancet. 2020;395(10223):514-523. doi:10.1016/S0140-6736(20)30154-9  93.Tian S, Hu N, Lou J, et al. Characteristics of COVID-19 infection in Beijing. J Infect. 2020;80(4):401-406. doi:10.1016/j.jinf.2020.02.018  94.Song R, Han B, Song M, et al. Clinical and epidemiological features of COVID-19 family clusters in Beijing, China [published online ahead of print, 2020 Apr 23]. J Infect. 2020;S0163-4453(20)30229-2. doi:10.1016/j.jinf.2020.04.018  95.Li Q, Guan X, Wu P, et al. Early Transmission Dynamics in Wuhan, China, of Novel Coronavirus-Infected Pneumonia. N Engl J Med. 2020;382(13):1199-1207. doi:10.1056/NEJMoa2001316  96.Ki M; Task Force for 2019-nCoV. Epidemiologic characteristics of early cases with 2019 novel coronavirus (2019-nCoV) disease in Korea. Epidemiol Health. 2020;42:e2020007. doi:10.4178/epih.e2020007  97.Xia XY, Wu J, Liu HL, Xia H, Jia B, Huang WX. Epidemiological and initial clinical characteristics of patients with family aggregation of COVID-19. J Clin Virol. 2020;127:104360. doi:10.1016/j.jcv.2020.104360  98.Sanche S, Lin YT, Xu C, Romero-Severson E, Hengartner N, Ke R. High Contagiousness and Rapid Spread of Severe Acute Respiratory Syndrome Coronavirus 2. Emerg Infect Dis. 2020;26(7):1470-1477. doi:10.3201/eid2607.200282  99.Cai J, Sun W, Huang J, Gamber M, Wu J, He G. Indirect Virus Transmission in Cluster of COVID-19 Cases, Wenzhou, China, 2020. Emerg Infect Dis. 2020;26(6):1343-1345. doi:10.3201/eid2606.200412  100.Pung R, Chiew CJ, Young BE, et al. Investigation of three clusters of COVID-19 in Singapore: implications for surveillance and response measures. Lancet. 2020;395(10229):1039-1046. doi:10.1016/S0140-6736(20)30528-6  101.Shen Q, Guo W, Guo T, et al. Novel coronavirus infection in children outside of Wuhan, China. Pediatr Pulmonol. 2020;55(6):1424-1429. doi:10.1002/ppul.24762  102.Wu WS, Li YG, Wei ZF, et al. Zhonghua Liu Xing Bing Xue Za Zhi. 2020;41(4):489-493. doi:10.3760/cma.j.cn112338-20200221-00139  103.Yang HY, Xu J, Li Y, et al. Zhonghua Liu Xing Bing Xue Za Zhi. 2020;41(5):623-628. doi:10.3760/cma.j.cn112338-20200223-00153  Zhang Y, Su X, Chen W, et al. Zhonghua Liu Xing Bing Xue Za Zhi. 2020;41(5):648-652. doi:10.3760/cma.j.cn112338-20200219-00121 |
| 4. Screened studies from Chinese database | 1.镇万源,蒋正文,王望才,等. 32例新型冠状病毒无症状感染者的临床特征分析[J]. 武汉大学学报(医学版), : 1-5.  2.李辉,段东辉,陈冰冰,等. 宁波市一起新型冠状病毒肺炎家庭聚集性疫情调查[J]. 预防医学, 2020, 32(9): 895-898.  3.白尧,陈志军,宋姝璇,等. 西安市一起新型冠状病毒肺炎家族聚集性疫情调查分析[J]. 山东大学学报(医学版), 2020, 58(10): 95-99.  4.王共飞,查涛,王毅,等. 新型冠状病毒无症状感染密切接触者的追踪检测[J]. 安徽预防医学杂志, 2020, 26(5): 368-370, 374.  5.张乐,张开义,马仲序,等. 昆明地区4例儿童新型冠状病毒感染临床特征分析[J]. 大理大学学报, 2020, 5(10): 11-15.  6.王宇红,张薇,张晓宇,等. 兰州市报告的首起新型冠状病毒肺炎家庭聚集性疫情调查[J]. 预防医学, 2020, 32(9): 891-894.  7.牛永亮,时靖峰,腾小宝,等. 100例新型冠状病毒感染者临床病例分析[J]. 川北医学院学报, 2020, 35(3): 547-550.  8.赵寒,熊宇,杨琳,等. 重庆市新型冠状病毒肺炎传染性分析[J]. 国际流行病学传染病学杂志, 2020, 47(3): 187-190.  9.郑隽,傅铁军,许静茹. 重庆市江北区COVID-19患者临床和实验室特征分析[J]. 重庆医科大学学报, 2020, 45(7): 942-948.  10.魏伟,袁成良,刘晓,等. 婴儿无症状感染新型冠状病毒伴粪便核酸检测持续阳性1例[J]. 解放军医学院学报, 2020, 41(3): 224-225, 228.  11.祝君兰,邓灵波,成官迅. 无症状COVID-19患者的胸部CT表现[J]. 医学信息, 2020, 33(16): 172-174.  12.刘碧瑶,戚小华,江敏,等. 浙江省境外输入新型冠状病毒肺炎病例流行特征分析[J]. 预防医学, 2020, 32(6): 550-554.  13.庞秋艳,李朋,李天忠,等. 一起高速服务区新型冠状病毒肺炎聚集性疫情调查[J]. 安徽预防医学杂志, 2020, 26(2): 130-132.  14.豆小文,王恩运,阚丽娟,等. 特异性IgM/IgG抗体检测筛查无症状新型冠状病毒感染者1例[J]. 临床检验杂志, 2020, 38(8): 581-582.  15.杨松,张力文,孙强中,等. 重庆市224例2019新冠病毒病的临床流行病学特点[C]//2020年全国结核病学术大会论文汇编, 2020: 552-566.  16.林君芬,吴梦娜,吴昊澄,等. 浙江省新型冠状病毒肺炎病例流行特征分析[J]. 预防医学, 2020, 32(3): 217-221, 225.  17.吕雪飞,方芳,张丹丹,等. 新型冠状病毒肺炎无症状感染者胸部CT征象[J]. 中国医学影像学杂志, 2020, 28(10): 730-733, 740.  18.曹安,蔡跃林,邱学斌,等. 苍南地区新型冠状病毒肺炎流行病学及临床特点分析[J]. 浙江医学, 2020, 42(10): 1055-1057.  19.赵磊,薛剑,王瑜玲,等. 石家庄地区输入性及本地发病的新型冠状病毒肺炎患者临床特征对比观察[J]. 山东医药, 2020, 60(15): 10-13.  20.谢仕兰,黄建华,刘珺,等. 广东省新型冠状病毒肺炎无症状感染者流行病学特征分析[J]. 中华流行病学杂志, 2020, 41(9): 1406-1410.  21.曹广文. 新型冠状病毒进化、相关流行特征和特异性防控中的关键问题[J]. 上海预防医学, 2020, 32(9): 697-703.  22.熊宇,赵寒,李勤. 2020年重庆市179例新型冠状病毒肺炎无症状感染者特征分析[J]. 重庆医学, : 1-5.  23.曹玉涵,宋素花. 对新型冠状病毒肺炎无症状感染者辨治思考[J]. 山东中医药大学学报, 2020, 44(5): 457-461.  24.贾蕾,王小莉,窦相峰,等. 北京市新型冠状病毒肺炎流行病学特征分析[J]. 首都公共卫生, 2020, 14(3): 120-123.  25.吴昊澄,丁哲渊,吴晨,等. 浙江省新型冠状病毒肺炎病例境外输入风险分析[J]. 预防医学, 2020, 32(6): 541-545.  26.郑思思,雍莉,兰莹,等. 四川省新型冠状病毒肺炎疫情防控工作阶段性思考[J]. 预防医学情报杂志, 2020, 36(10): 1265-1267.  27.胡小琦,彭焕文,刘志勇,等. 一起无症状感染者引起的新型冠状病毒肺炎聚集性疫情调查分析[J]. 预防医学情报杂志, 2020, 36(7): 800-803.  28.刘志荣,苏斌,吴家兵,等. 一起新型冠状病毒肺炎聚集性疫情调查[J]. 中华疾病控制杂志, 2020, 24(5): 512-515, 522.  29.覃常宇,邓茂铭,罗涛,等. 一起无症状新型冠状病毒感染者引起的聚集性疫情[J]. 河南预防医学杂志, 2020, 31(11): 857-859, 871.  30.周良君,温浩瑄,陈果,等. 一起无症状感染者引起的新型冠状病毒肺炎疫情的跟踪检测报告[J]. 医学动物防制, 2020, 36(11): 1021-1024.  31.丁焕,钱静娟,高雨蒙,等. 无锡市新型冠状病毒肺炎无症状感染者的传播风险分析[J]. 国际流行病学传染病学杂志, 2020, 47(5): 465-468.  32.陈敏红,贾海梅,王瀚炜,等. 福建省福州市新型冠状病毒肺炎病例及其密切接触者感染情况分析[J]. 疾病监测, 2020, 35(7): 608-612.  33.黄小梅,黄婷,袁珩,等. 四川省新型冠状病毒肺炎无症状感染者流行病学特征分析[J]. 现代预防医学, 2020, 47(18): 3279-3283.  34.陈世囝,魏玉凤,焦英杰,等. 新型冠状病毒感染无症状患儿诊治报告1例[J]. 中国民间疗法, 2020, 28(14): 1-3.  35.王德华,郑欢伟,王建民,等. 石家庄地区45例COVID-19患者临床特征分析[J]. 传染病信息, 2020, 33(5): 416-418, 425.  36.叶莹,范威,王文华,等. 新型冠状病毒肺炎聚集性疫情中无症状感染者与确诊患者的流行差异[J]. 中国感染控制杂志, 2020, 19(6): 492-497.  37.马蒙蒙,马晓薇,吴燕,等. 广州市新型冠状病毒肺炎疫情流行特征分析[J]. 华南预防医学, 2020, 46(4): 380-384.  38.马兰,柳赟昊,张玲,等. 武汉市一起新冠确诊病例出院后家庭再现病例调查[J]. 中华疾病控制杂志, 2020, 24(11): 1343-1347.  39.宋翊,谢鑫,罗晓琼,等. 新型冠状病毒无症状感染者50例临床特点分析[J]. 中国中医急症, 2020, 29(11): 1885-1887, 1933.  40.王克顺,叶志豪,张定. 平阳县一起新型冠状病毒肺炎聚集性疫情调查[J]. 预防医学, 2020, 32(3): 222-225.  41.陈翠霞,张晓丽. 2020年临沂市新型冠状病毒肺炎病例情况分析[J]. 预防医学论坛, 2020, 26(10): 775-777.  42.范威,叶莹,王文华,等. 新型冠状病毒肺炎多代聚集性病例流行特征调查[J]. 中国人兽共患病学报, 2020, 36(5): 377-382.  43.胡永峰,刘立平,姚喜清,等. 武汉市某区新型冠状病毒肺炎密切接触者感染与发病流行病学特征分析[J]. 现代预防医学, 2020, 47(21): 3993-3997.  44.白尧,吉兆华,张辉,等. 西安市新型冠状病毒肺炎疫情流行特征[J]. 中华疾病控制杂志, 2020, 24(5): 567-572.  45.阳雅兰,李林洪,李长凤,等. 重庆市一起新型冠状病毒肺炎家庭聚集性疫情调查分析[J]. 中国公共卫生, 2020, 36(3): 285-288.  46.李应强,余丽丽,刘萍,等. 1例输入性家庭聚集性新型冠状病毒肺炎疫情传播路径解析[J]. 寄生虫病与感染性疾病, 2020, 18(3): 137-140.  47.余夏,周莹,阳文辉,等. 108例住院患者2019新型冠状病毒核酸检测结果分析[J]. 中国临床新医学, 2020, 13(5): 448-452.  48.周巾力,黄河秋,胡艳,等. 河南省信阳市51起新型冠状病毒肺炎聚集性疫情流行病学特征[J]. 中国热带医学, 2020, 20(11): 1078-1081.  49.邓志强,夏文,范义兵,等. 南昌市一起新型冠状病毒肺炎聚集性疫情传播链分析[J]. 中华流行病学杂志, 2020, 41(9): 1420-1423.  50.彭焕文,胡小琦,潘霞,等. 四川省达州市42例新型冠状病毒肺炎病例的流行特征[J]. 中国热带医学, 2020, 20(11): 1066-1068.  51.李福兴,谢可心,张玉琳,等. 云南省新型冠状病毒肺炎流行情况初步分析[J]. 大理大学学报, 2020, 5(8): 1-6.  52.周仁彬,陈益香,林创兴,等. 无症状伴肺部影像学改变的新型冠状病毒肺炎孕妇一例[J]. 中华围产医学杂志, 2020, (3): 166-167.  53.倪晓媚,柯珍,季小微,等. 青田县一起境外输入新型冠状病毒肺炎聚集性疫情分析[J]. 预防医学, 2020, 32(7): 678-681.  54.黄贤平,谢树华,江丽玲,等. 赣南地区儿童新型冠状病毒感染肺炎的临床特征及影像学特点分析[J]. 赣南医学院学报, 2020, 40(3): 239-242.  55.李艳艳,张俊杰,王慧晶,等. 平顶山市一起新型冠状病毒肺炎聚集性病例调查分析[J]. 医学动物防制, 2021, 37(1): 71-74.  56.王玲,曹海霞,张玲,等. 淄博市一起新型冠状病毒肺炎家族聚集性疫情调查分析[J]. 山东大学学报(医学版), 2020, 58(10): 100-104, 111.  57.刘卫,朱韩武,刘勋,等. 新型冠状病毒肺炎家庭聚集性疫情传染源和传播链调查分析[J]. 华南预防医学, 2020, 46(5): 506-510.  58.董玉颖,范前东,王月萍,等. 江苏省扬州市新型冠状病毒肺炎患者核酸检测结果分析[J]. 实用临床医药杂志, 2020, 24(5): 6-9.  59.陈军,王险峰,张培发. 新型冠状病毒感染无症状患儿20例临床分析[J]. 中国当代儿科杂志, 2020, 22(5): 414-418.  60.王俊,王亦聪,王吉国,等. 宜城市2020年新型冠状病毒肺炎流行特征分析[J]. 华南预防医学, 2020, 46(5): 511-513.  61.孙佰红,井丽,李鑫,等. 辽宁省32例新型冠状病毒肺炎无症状感染者流行病学特征[J]. 中国热带医学, 2020, 20(10): 922-925.  62.刘毅,邹于生,陈慧明,等. 江西某县一起家庭聚集性新型冠状病毒感染事件调查分析[J]. 实用临床医学, 2020, 21(3): 83-85.  63.高文静,郑可,柯骥,等. 新型冠状病毒肺炎无症状感染相关研究进展[J]. 中华流行病学杂志, 2020, 41(7): 990-993.  64.刘志刚,胡春生,邹永庚,等. 一起新型冠状病毒肺炎聚集性疫情流行病学调查分析[J]. 实用预防医学, 2020, 27(11): 1304-1307.  65.陈奕,王爱红,易波,等. 宁波市新型冠状病毒肺炎密切接触者感染流行病学特征分析[J]. 中华流行病学杂志, 2020, (5): 667-671.  66.胡世雄,徐巧华,罗垲炜,等. 湖南省新型冠状病毒肺炎感染者流行病学特征分析[J]. 实用预防医学, 2020, 27(4): 385-388.  67.魏建华,高小娜,郭蕾,等. 宁夏定点医院收治68例确诊新型冠状病毒感染肺炎患者临床症状分析[J]. 宁夏医学杂志, 2020, 42(3): 265-266.  68.黄瑶,徐娅雯,王艳,等. 婴儿SARS-CoV-2无症状感染1例报道[J]. 检验医学, 2020, 35(10): 1079-1082.  69.卢伟霞,王松强,王小丽,等. 郑州市新型冠状病毒肺炎无症状感染者调查[J]. 预防医学, 2020, 32(12): 1277-1278.  70.姚莉,王菁,赵晶晶,等. 孕晚期合并无症状新型冠状病毒感染一例[J]. 中华围产医学杂志, 2020, (3): 229-230.  71.马婉婉,苏斌,吴家兵,等. 安徽地区一起新型冠状病毒肺炎家庭聚集性疫情调查[J]. 中国公共卫生, 2020, 36(3): 277-281.  72.周仁彬,陈益香,林创兴,等. 无症状伴肺部影像学改变的新型冠状病毒感染孕妇一例[J]. 中华围产医学杂志, 2020, 23(3): 0-0.  73.王晟,刘兴态,秦军,等. 738例新型冠状病毒肺炎病例密切接触者核酸筛查结果分析[J]. 中国感染控制杂志, 2020, 19(4): 297-300.  74.李锦成,徐勤,王艳,等. 江苏省扬州市新型冠状病毒肺炎无症状感染者的特征分析[J]. 实用临床医药杂志, 2020, 24(5): 10-13.  75.赵建华,张晓琴,柴军,等. 无症状感染转确诊新型冠状病毒肺炎胸部CT分析[J]. 生物医学工程与临床, 2020, 24(6): 672-677.  76.姚莉,王菁,赵晶晶,等. 孕晚期合并无症状新型冠状病毒感染一例[J]. 中华围产医学杂志, 2020, 23(3): 169-171.  77.孙倩莱,李作超,谭夏林,等. 一起新型冠状病毒肺炎聚集性疫情调查[J]. 实用预防医学, 2020, 27(4): 389-392.  78.唐琳,罗强,刘军,等. 衡阳市新型冠状病毒肺炎流行病学特征分析及防控措施评估[J]. 实用预防医学, 2020, 27(8): 912-916.  79.吴群,曾小平,王明昌,等. 海口市一起潜伏期新型冠状病毒肺炎病例引起的聚集性疫情分析[J]. 预防医学, 2020, 32(7): 670-673.  80.白尧,孙亚辉,刘继锋,等. 陕西省一起新型冠状病毒肺炎聚集性疫情调查分析[J]. 上海预防医学, : 1-6.  81.高志霞,周健,姜艳艳,等. 院内感染为主引起的多家庭新冠肺炎聚集性疫情调查[J]. 中国热带医学, 2020, 20(7): 673-675.  82.高海燕,侯培生. 某县儿童感染新型冠状病毒的流行病学分析[J]. 中国药物与临床, 2020, 20(15): 2629-2631.  83.邓明菊,边绍勇,廖强,等. 凉山州1起新型冠状病毒肺炎家庭聚集性疫情调查分析[J]. 预防医学情报杂志, 2020, 36(10): 1260-1264.  84.韩若东,马景贺,艾辉,等. 108例新型冠状病毒肺炎患者的临床特征分析[J]. 实用预防医学, 2020, 27(9): 1040-1043.  85.周丽君,王小娟,程秀伟,等. 四川省新型冠状病毒肺炎聚集性疫情流行特征分析[J]. 现代预防医学, 2020, 47(21): 3867-3870.  86.谢燕湘,邓海斌,谢朝梅,等. 常德市重点人群新型冠状病毒感染流行病学调查分析[J]. 实用预防医学, 2020, 27(7): 776-779.  87.高海军,张颋,许光荣,等. 四川省甘孜藏族自治州新型冠状病毒肺炎病例流行病学特征分析[J]. 疾病监测, 2020, 35(9): 793-797.  88.丁克琴,易波,陈奕,等. 浙江省宁波市新型冠状病毒肺炎聚集性疫情流行病学分析[J]. 中国公共卫生, 2020, 36(4): 498-502.  89.刘阳,刘润友,王卓,等. 四川省新型冠状病毒肺炎流行病学特征分析[J]. 预防医学情报杂志, 2020, 36(7): 793-799.  90.董文逸,黄高燕,谢周华,等. 55例新型冠状病毒肺炎患者临床特征[J]. 中国热带医学, 2020, 20(10): 959-961.  91.张红杰,邢雅素,李永刚. 天津市B区一起家庭聚集性新型冠状病毒肺炎疫情调查分析[J]. 实用预防医学, 2020, 27(9): 1037-1039.  92.田丽丽,钱城,辛若雷,等. 一起境外输入新型冠状病毒肺炎家庭聚集性疫情流行病学调查和诊疗分析[J]. 首都公共卫生, 2020, 14(3): 132-136.  93.廖芳芳,蔡朝阳,胡明霞,等. 新型冠状病毒无症状感染孕妇1例的院感防控实践[J]. 安徽卫生职业技术学院学报, 2020, 19(3): 9-10, 13.  94. 什么是新型冠状病毒无症状感染者?[J]. 中国卫生信息管理杂志, 2020, 17(2): 162.  95.李映霞,谭文艳,刘勋,等. 湖南省郴州市新型冠状病毒肺炎流行病学特征分析[J]. 上海预防医学, 2020, 32(9): 716-721.  96.张栋梁,易波,陈奕,等. 宁波市新型冠状病毒肺炎病例流行特征分析[J]. 预防医学, 2020, 32(4): 330-333.  97.徐军,李静娟. 一起新型冠状病毒无症状感染者相关聚集性疫情的流行病学调查[J]. 实用预防医学, 2020, 27(7): 787-789.  98.白尧,陈志军,陈保忠,等. 西安市新型冠状病毒肺炎聚集性疫情流行特征[J]. 中国热带医学, 2020, 20(9): 853-856, 866.  99.吴艳玲,魏峰,李琦,等. 承德市新型冠状病毒肺炎流行病学特征分析[J]. 河北医学, 2020, 26(6): 887-892.  100.刘勋,刘飞,凡琴,等. 郴州地区一起无症状感染者引起新型冠状病毒肺炎家庭聚集性疫情[J]. 中国公共卫生, 2020, 36(3): 282-284.  101.杨静,董剑,杨春燕,等. 重庆大足区新型冠状病毒肺炎患者出院后病毒核酸随访结果[J]. 病毒学报, 2020, 36(4): 549-553.  102.李游江,胡瑛瑛,张晓东,等. 七例2019冠状病毒病(COVID-19)普通型患者出院后病毒核酸随访结果[J]. 浙江大学学报(医学版), 2020, 49(2): 270-274.  103.陈露,魏文洲,刘远健,等. COVID-19恢复期MSCT征象及核酸复阳与非复阳MSCT征象对比分析[J]. 放射学实践, 2020, 35(11): 1375-1379.  104.维超,孙贵银,李峰,等. 3例新型冠状病毒肺炎出院患者病毒核酸检测复阳原因探讨[J]. 国际检验医学杂志, 2020, 41(20): 2550-2554.  105.乐,张崇唯,曹钰,等. 免疫功能与新型冠状病毒肺炎康复患者核酸检测结果复阳的关系研究[J]. 国际检验医学杂志, 2020, 41(18): 2251-2254.  106.李玉柱,师凌昊,梁冬,等. 症状好转后影像加重复检阳性新型冠状病毒肺炎1例思考[J]. 中华医院感染学杂志, 2020, 30(17): 2588-2591.  107.曾金彩,黄淑芬,邓小玲,等. COVID-19出院患者“复阳”情况及原因分析[J]. 华南预防医学, 2020, 46(5): 514-518.  108.胡雯雯,王梅芳,常婵,等. 新型冠状病毒肺炎患者核酸检测“复阳”的特点及危险因素分析（英文）[J]. 生命科学研究, 2020, 24(6): 431-441.  109.姚令辉,苏小可,段良松,等. 郴州市新型冠状病毒肺炎确诊病例出院复阳情况报告[J]. 湘南学院学报(医学版), 2020, 22(1): 47-48.  110.林红东,蓝博文,周玉祥,等. COVID-19在不同时期的胸部CT表现及出院后核酸复阳肺内病灶变化特点[J]. 分子影像学杂志, 2020, 43(4): 705-708.  111.李泉,张浩,邓斯予,等. 新型冠状病毒肺炎恢复期病毒核酸检测复阳患者与阴性患者外周血淋巴细胞亚群的表达及形态学特征的比较研究[J]. 重庆医学, 2020, 49(19): 3151-3155.  112.孔祥亘,布学慧,王彬,等. 新型冠状病毒病患者病毒核酸反复阳性超60天2例报告[J]. 山东大学学报(医学版), 2020, 58(10): 117-119, 126.  113.吕客,李英姿,刘绍龙,等. 中医药治疗新型冠状病毒感染愈后复阳3例[J]. 中医药临床杂志, 2020, 32(8): 1424-1427.  114.林晓敏,刘杼升,陈世准. 新冠病毒感染患者23例临床病例分析[J]. 包头医学院学报, 2020, 36(10): 20-23.  115.余昪昪,杨玉姣. 新型冠状病毒核酸检测“复阳”1例[J]. 中国感染与化疗杂志, 2020, 20(6): 694-695.  116.郑倩文,车峰远,李爱香,等. 41例新型冠状病毒肺炎患者临床特征及“复阳”结果分析[J]. 检验医学与临床, 2020, 17(23): 3510-3515.  117.智深深,许毅,陈耀凯,等. 五例新型冠状病毒肺炎确诊患者出院后核酸检测复阳的病例分析[J]. 中华检验医学杂志, 2020, 43(9): 923-926.  118.单迎光,郜旌宏,李风祥,等. 182例非重症型新型冠状病毒肺炎患者临床观察及合并心血管疾病的情况分析[J]. 中国循环杂志, 2020, 35(11): 1097-1102.  119.王宇,刘岩岩,汤艳芬,等. 新型冠状病毒肺炎患者出院隔离观察期核酸复阳情况分析[J]. 实用心脑肺血管病杂志, 2020, 28(12): 5-8.  120.赵莹,吴伟慎,何海艳,等. 天津市新型冠状病毒肺炎确诊病例治愈出院后核酸阳转情况分析[J]. 第三军医大学学报, 2020, 42(9): 879-882.  121.施丽泳,蔡艳萍,张祎祥,等. 新型冠状病毒肺炎核酸复阳患者与未复阳患者临床特征比较[J]. 中华结核和呼吸杂志, 2020, 43(12): 1066-1070.  122.艾香英,傅晓霞,林路平,等. 30例新型冠状病毒核酸复阳返院患者的病例特点[J]. 中国感染控制杂志, 2020, 19(7): 591-596.  123.郑隽,傅铁军,许静茹. 重庆市江北区COVID-19患者临床和实验室特征分析[J]. 重庆医科大学学报, 2020, 45(7): 942-948.  124.周灵,刘威,方媛媛,等. 出院后新型冠状病毒核酸复阳的新型冠状病毒肺炎患者11例临床特征分析[J]. 临床内科杂志, 2020, 37(3): 242-244.  125.宋曙,石雨涵,唐海成,等. 关于新型冠状病毒肺炎患者出院后核酸检测SARS-CoV-2 RNA“复阳”原因及对策分析[J]. 中国微生态学杂志, 2020, 32(7): 786-788.  126.黄加美,袁超,黄德扬,等. 广州地区新型冠状病毒肺炎患者核酸“复阳”的CT表现和临床特征[J]. 实用医学杂志, 2020, 36(21): 2889-2893.  127.李盼盼,吴彪,巫华志,等. 61例新型冠状病毒肺炎患者出院后随访调查分析[J]. 中国热带医学, 2020, 20(10): 976-979.  128.罗东霞,刘大凤,刘亚玲,等. 成都市1例输入性新型冠状病毒肺炎病例诊治及两次复阳过程探讨[J]. 中国热带医学, 2020, 20(12): 1216-1219.  129.赵本南,刘大凤,刘亚玲,等. 普通型新型冠状病毒肺炎患者出院后复诊结果分析[J]. 成都医学院学报, : 1-10.  130.代艳,唐宁宁,徐帆,等. 广西儿童新型冠状病毒肺炎11例临床分析[J]. 中国临床新医学, 2020, 13(11): 1149-1152.  131.宋惠雯,曾祥铨,连豫苞,等. 14例新型冠状病毒肺炎患者的临床特点及重型患者的救治体会[J]. 现代医药卫生, 2020, 36(23): 3713-3716, 3720.  132.卓丽,魏芳芳,周建群,等. 新型冠状病毒肺炎患者34例转归影响因素[J]. 实用医学杂志, 2020, 36(14): 1861-1865.  133.胡星星,倪岚,陈毅斐,等. 新型冠状病毒肺炎患者恢复期核酸检测复阳的临床分析[J]. 武汉大学学报(医学版), : 1-5.  134.黄慧琴,谷斌,曹晓英,等. 39例新型冠状病毒肺炎患者临床特征分析[J]. 传染病信息, 2020, 33(4): 312-316.  135.罗纯,温学良,谭颖,等. 广州地区新型冠状病毒肺炎出院病例核酸再次阳性的临床特征[J]. 广东医学, 2020, 41(13): 1297-1301.  136.杨金燕,陈元宾,夏婷婷,等. 1例轻型新型冠状病毒肺炎愈后“复阳”病例的诊疗实践及其影响因素[J]. 中华医院感染学杂志, 2020, 30(19): 2913-2917.  137.梁超,费敏,凌晓敏,等. 普通型新型冠状病毒肺炎患者咽拭子病毒核酸检测结果复阳情况分析[J]. 中国临床医学, 2020, 27(3): 366-368.  138.阚小华,李纯,孙加奎,等. 鼻咽拭子核酸检测连续阴性次数对COVID-19出院患者复阳率的影响[J]. 江苏医药, 2020, 46(8): 843-845.  139.刘晓鹏,王红宇,张思森,等. 新冠肺炎出院患者核酸“复阳”现象的临床评析[J]. 河南医学研究, 2020, 29(9): 1537-1541.  140.李泉,张浩,邓斯予,等. COVID-19恢复期病毒核酸检测复阳与阴性患者淋巴细胞亚群及形态学特征比较研究[J]. 重庆医学, : 1-6.  141.李小玲,王晓光,李兵,等.新冠肺炎超长时间排毒1例诊治分析[J]. 人民军医, 2020, 63(5): 502-504, 508. |

# Completeness of searching terms

Infectious Disease Incubation Period OR incubation OR incubation period OR period OR asymptomatic OR presymptomatic OR preclinic period OR recurrent positive OR reinfection OR symptomatic AND COVID-19 OR 2019 novel coronavirus disease OR COVID19 OR COVID-19 pandemic OR SARS-CoV-2 infection OR COVID-19 virus disease OR 2019 novel coronavirus infection OR 2019-nCoV infection OR coronavirus disease 2019 OR coronavirus disease-19 OR 2019-nCoV disease OR COVID-19 virus infection

**Previous Studies reported large percentiles of the COVID-19 incubation period distribution**

| Author | Location | Sample | Data acquisition time | Publication time | Percentiles of incubation period in days(95% *Confidence Interval)* | | |
| --- | --- | --- | --- | --- | --- | --- | --- |
|  |  |  |  |  | p95 | P97.5 | P99 |
| Yu etal [7] | Shanghai, China | 132 | 19/Feb/2020 | 4/Apr/2020 | 16 |  | 20.4 |
| Bi etal [8] | Shenzhen, China | 183 | 14/Jan/2020- 12/Feb/2020 | 27/Apr/2020 | 14 (12.2‒15.9) |  |  |
| Lai etal [9] | Hongkong, China | 100 | 18/Jan/2020- 2/Mar/2020 | 20/May/2020 | 14 (13.1‒15.3) |  |  |
| Qin etal [10] | Many counties in the world | 1084 | 20/Jan/2020- 29/Feb/2020 | 2/July/2020 | 16.32 (15.62‒-17.04) |  | 20.31 (19.15‒21.47) |
| Lu etal [11] | Mainland China | 1158 | 1/Jan/2020- 11/Feb/2020 | 14/July/2020 | 15.1 (14.4‒15.7) |  | 18.7 (17.6‒19.8) |
| Wu etal [12] | Zhuhai, china | 48 | Jan/2020- Feb/2020 | 11/May/2020 | 15.3 (10.4‒21.1) |  |  |
| Zhang etal [13] | Mainland China | 49 | 19/Jan/2020- 17/Feb/2020 | 2/Apr/2020 | 10.5 |  |  |
| Hu etal [14] | Hunan, china | 268 | 23/Feb/2020- 2/Apr/2020 | 2/Nov/2020 |  | 16.6 |  |
| Li etal [15] | Wuhan, China | 10 | 22/Jan/2020 | 29/Jan/2020 | 12.5 (9.2‒18) |  |  |
| Yang etal [16] | Shiyan, China | 178 | 20/Jan/2020- 29/Feb/2020 | 16/June/2020 | 13.7 (12.5‒14.9) | 15 |  |
| Ma etal [17] | China, Japan, Singapore | 587 | 20/Jan/2020- 12/Feb/2020 | 1/Oct/2020 | 17 |  |  |
| Bi etal [18] | Shenzhen, China | 183 | 14/Jan/2020- 12/Feb/2020 | preprint | 14 (12.2‒15.9) |  |  |
| Backer etal [19] | Wuhan, China | 88 | 20/Jan/2020- 28/Jan/2020 | 6/Feb/2020 | 10.3 (8.6‒14.1) |  |  |
| Linton etal [20] | Wuhan, China | 158 | -31/Jan/2020 | 17/Feb/2020 | 12 (9.8‒15.6) |  |  |
| Lauer etal [21] | Wuhan, China | 181 | 4/Jan/2020- 24/Feb/2020 | 10/Mar/2020 |  | 11.5 (8.2‒15.6) |  |
| Leung etal [22] | Wuhan, China | 175 | 20/Jan/2020- 12/Feb/2020 | 18/Mar/2020 | 14.6 (12.1‒17.1) |  |  |
| Shi etal [23] | Wuxi, China | 46 | 18/Jan/2020- 2/Mar/2020 | 13/Oct/2020 | 12 (10.09‒13.91) |  |  |

**Table S3.** Published studies related to large percentiles estimates of the COVID-19 incubation period distribution.

**References**

1. National Health Commission of China. Prevention and Control of Novel Coronavirus Pneumonia, 6th edn (2020).
2. Kimball A, Hatfield KM, Arons M, James A, Taylor J, Spicer K, et al. Asymptomatic and Presymptomatic SARS-CoV-2 Infections in Residents of a Long-Term Care Skilled Nursing Facility - King County, Washington, March 2020. MMWR Morb Mortal Wkly Rep. 2020;69(13):377-381. doi: 10.15585/mmwr.mm6913e1.
3. Lan L, Xu D, Ye G, Xia C, Wang S, Li Y, et al. Positive RT-PCR Test Results in Patients Recovered From COVID-19. JAMA. 2020;323(15):1502-1503. doi: 10.1001/jama.2020.2783.
4. Tang X, Zhao S, He D, Yang L, Wang MH, Li Y, et al. Positive RT-PCR tests among discharged COVID-19 patients in Shenzhen, China. Infect Control Hosp Epidemiol. 2020;41(9):1110-1112. doi: 10.1017/ice.2020.134.
5. Lessler J, Reich NG, Brookmeyer R, Perl TM, Nelson KE, Cummings DA. Incubation periods of acute respiratory viral infections: a systematic review. Lancet Infect Dis. 2009;9(5):291-300. doi: 10.1016/S1473-3099(09)70069-6.
6. Linton NM, Kobayashi T, Yang Y, Hayashi K, Akhmetzhanov AR, Jung SM, et al. Incubation Period and Other Epidemiological Characteristics of 2019 Novel Coronavirus Infections with Right Truncation: A Statistical Analysis of Publicly Available Case Data. J Clin Med. 2020;9(2):538. doi: 10.3390/jcm9020538.
7. Bi Q, Wu Y, Mei S, Ye C, Zou X, Zhang Z, et al. Epidemiology and transmission of COVID-19 in 391 cases and 1286 of their close contacts in Shenzhen, China: a retrospective cohort study. Lancet Infect Dis. 2020;20(8):911-919. doi: 10.1016/S1473-3099(20)30287-5.
8. Lai CKC, Ng RWY, Wong MCS, Chong KC, Yeoh YK, Chen Z, et al. Epidemiological characteristics of the first 100 cases of coronavirus disease 2019 (COVID-19) in Hong Kong Special Administrative Region, China, a city with a stringent containment policy. Int J Epidemiol. 2020;49(4):1096-1105. doi: 10.1093/ije/dyaa106.
9. Qin J, You C, Lin Q, Hu T, Yu S, Zhou XH. Estimation of incubation period distribution of COVID-19 using disease onset forward time: A novel cross-sectional and forward follow-up study. Sci Adv. 2020;6(33):eabc1202. doi: 10.1126/sciadv.abc1202.
10. Lu QB, Zhang Y, Liu MJ, Zhang HY, Jalali N, Zhang AR,et al. Epidemiological parameters of COVID-19 and its implication for infectivity among patients in China, 1 January to 11 February 2020. Euro Surveill. 2020;25(40):2000250. doi: 10.2807/1560-7917.ES.2020.25.40.2000250.
11. Wu J, Huang Y, Tu C, Bi C, Chen Z, Luo L, et al. Household Transmission of SARS-CoV-2, Zhuhai, China, 2020. Clin Infect Dis. 2020;71(16):2099-2108. doi: 10.1093/cid/ciaa557.
12. Zhang J, Litvinova M, Wang W, Wang Y, Deng X, Chen X, et al. Evolving epidemiology and transmission dynamics of coronavirus disease 2019 outside Hubei province, China: a descriptive and modelling study. Lancet Infect Dis. 2020 Jul;20(7):793-802. doi: 10.1016/S1473-3099(20)30230-9.
13. Hu S, Wang W, Wang Y, Litvinova M, Luo K, Ren L, et al. Infectivity, susceptibility, and risk factors associated with SARS-CoV-2 transmission under intensive contact tracing in Hunan, China. medRxiv [Preprint]. 2020:2020.07.23.20160317. doi: 10.1101/2020.07.23.20160317.
14. Li Q, Guan X, Wu P, Wang X, Zhou L, Tong Y, et al. Early Transmission Dynamics in Wuhan, China, of Novel Coronavirus-Infected Pneumonia. N Engl J Med. 2020;382(13):1199-1207. doi: 10.1056/NEJMoa2001316.
15. Yang L, Dai J, Zhao J, Wang Y, Deng P, Wang J. Estimation of incubation period and serial interval of COVID-19: analysis of 178 cases and 131 transmission chains in Hubei province, China. Epidemiol Infect. 2020;148:e117. doi: 10.1017/S0950268820001338.
16. Ma S, Zhang J, Zeng M, Yun Q, Guo W, Zheng Y, et al. Epidemiological Parameters of COVID-19: Case Series Study. J Med Internet Res. 2020;22(10):e19994. doi: 10.2196/19994.
17. Bi Q, Wu Y, Mei S, Ye C, Zou X, Zhang Z, et al. Epidemiology and transmission of COVID-19 in 391 cases and 1286 of their close contacts in Shenzhen, China: a retrospective cohort study. Lancet Infect Dis. 2020;20(8):911-919. doi: 10.1016/S1473-3099(20)30287-5.
18. Backer JA, Klinkenberg D, Wallinga J. Incubation period of 2019 novel coronavirus (2019-nCoV) infections among travellers from Wuhan, China, 20-28 January 2020. Euro Surveill. 2020;25(5):2000062. doi: 10.2807/1560-7917.ES.2020.25.5.2000062.
19. Linton NM, Kobayashi T, Yang Y, Hayashi K, Akhmetzhanov AR, Jung SM, et al. Incubation Period and Other Epidemiological Characteristics of 2019 Novel Coronavirus Infections with Right Truncation: A Statistical Analysis of Publicly Available Case Data. J Clin Med. 2020;9(2):538. doi: 10.3390/jcm9020538.
20. Lauer SA, Grantz KH, Bi Q, Jones FK, Zheng Q, Meredith HR, et al. The Incubation Period of Coronavirus Disease 2019 (COVID-19) From Publicly Reported Confirmed Cases: Estimation and Application. Ann Intern Med. 2020;172(9):577-582. doi: 10.7326/M20-0504. Epub 2020 Mar 10.
21. Leung C. The difference in the incubation period of 2019 novel coronavirus (SARS-CoV-2) infection between travelers to Hubei and nontravelers: The need for a longer quarantine period. Infect Control Hosp Epidemiol. 2020;41(5):594-596. doi: 10.1017/ice.2020.81.
22. Shi P, Gao Y, Shen Y, Chen E, Chen H, Liu J, et al. Characteristics and evaluation of the effectiveness of monitoring and control measures for the first 69 Patients with COVID-19 from 18 January 2020 to 2 March in Wuxi, China. Sustain Cities Soc. 2021;64:102559. doi: 10.1016/j.scs.2020.102559.
